# Patient-specific heart simulation can identify non-responders to cardiac resynchronization therapy

**DOI:** 10.1007/s00380-020-01577-1

**Published:** 2020-03-12

**Authors:** Akihiro Isotani, Kazunori Yoneda, Takashi Iwamura, Masahiro Watanabe, Jun-ichi Okada, Takumi Washio, Seiryo Sugiura, Toshiaki Hisada, Kenji Ando

**Affiliations:** 1 Department of Cardiovascular Medicine, Kokura Memorial Hospital, Asano 3-2-1, Kokurakita-ku, Kitakyushu, Fukuoka 802-8555 Japan; 2 Healthcare System Unit, Fujitsu Ltd, Ota-ku, Kamata, 144-8588 Japan; 3 Future Center Initiative, The University of Tokyo, Wakashiba 178-4-4, Kashiwa, Chiba 277-0871 Japan; 4 UT-Heart Inc. Nozawa, 3-25-8, Setagaya, Tokyo 154-0003 Japan; 5 Future Center #304, Wakashiba 178-4-4, Kashiwa, Chiba 277-0871 Japan

**Keywords:** Cardiac resynchronization therapy, Non-responders, Bi-ventricular pacing, Patient-specific heart model, dP/dtmax

## Abstract

To identify non-responders to cardiac resynchronization therapy (CRT), various biomarkers have been proposed, but these attempts have not been successful to date. We tested the clinical applicability of computer simulation of CRT for the identification of non-responders. We used the multi-scale heart simulator “UT-Heart,” which can reproduce the electrophysiology and mechanics of the heart based on a molecular model of the excitation–contraction mechanism. Patient-specific heart models were created for eight heart failure patients who were treated with CRT, based on the clinical data recorded before treatment. Using these heart models, bi-ventricular pacing simulations were performed at multiple pacing sites adopted in clinical practice. Improvement in pumping function measured by the relative change of maximum positive derivative of left ventricular pressure (%Δd*P*/d*t*_max_) was compared with the clinical outcome. The operators of the simulation were blinded to the clinical outcome. In six patients, the relative reduction in end-systolic volume exceeded 15% in the follow-up echocardiogram at 3 months (responders) and the remaining two patients were judged as non-responders. The simulated %Δd*P*/d*t*_max_ at the best lead position could identify responders and non-responders successfully. With further refinement of the model, patient-specific simulation could be a useful tool for identifying non-responders to CRT.

**Electronic supplementary material:**

The online version of this article (10.1007/s00380-020-01577-1) contains supplementary material, which is available to authorized users.

## Introduction

The safety and efficacy of cardiac resynchronization therapy (CRT) have been proved by a number of landmark studies [1–3], but these studies also revealed that a significant proportion of patients indicated for CRT do not respond to this invasive and expensive treatment (non-responders) [4]. To reduce the number of non-responders, efforts have been made toward proper positioning of pacing electrodes, optimization of the stimulation intervals, and the development of novel pacemaker devices. However, the appropriate selection of patients remains key to solving this problem [5].

Because the mechanical dyssynchrony resulting from dyssynchronous electrical activation can be an important target of therapy, various echocardiographic indices have been evaluated regarding whether they have diagnostic value additional to electrocardiogram (ECG) biomarkers. To date, none of the large trials have reported positive findings [6, 7]. Patient characteristics, including ischemic etiology and the existence of scar tissue, are believed to affect the therapeutic response, but randomized controlled trials show controversial results [5]. Furthermore, the requirement of cardiac magnetic resonance imaging (MRI) or nuclear imaging for the accurate evaluation of scar burden may hinder its use in daily clinical practice [8]. Accordingly, only ECG indices including QRS width and morphology, such as left bundle branch block, are adopted as the criteria for class I indication in current guidelines [9, 10].

Computer simulation of the heart has emerged as a novel tool in cardiology research and its applications for CRT have already been reported [11]. Such applications include not only studies for understanding the mechanism of CRT and/or searches for optimal pacing strategies using standard heart models but also those attempting to predict therapeutic outcomes with patient-specific models, thus aiming at an alternative approach to patient selection [12, 13].

Among these, the University of Tokyo heart simulator is a multi-scale, multi-physics heart simulator, in which the function of the heart, including patient-specific mechanics and electrophysiology, is reproduced in a three-dimensional heart model based on the molecular mechanism of the excitation–contraction coupling process [14–18]. Okada et al. applied this simulation model to patient-specific CRT simulation to show that the simulated changes in ventricular function, measured by the maximum rate of rise in ventricular pressure (d*P*/d*t*_max_), correlated well with the clinically observed changes in ejection fraction (EF) in a retrospective study [19]. Because, however, information on the actual pacing site was utilized, this approach cannot be translated to the prediction of non-responders in practice.

In this study, we extended this approach to examine whether the UT-Heart heart simulator can identify non-responders to CRT based only on the clinical data recorded before the CRT implantation. The results demonstrated that the heart simulator has potential to provide additional value to the current guidelines for the selection of CRT candidates.

## Materials and methods

### Study patients

Among the heart failure patients treated with CRT at the Kokura Memorial Hospital between April and December 2016, eight patients, whose cardiac computer tomography (CT) images were recorded before implantation, were enrolled in this study (age 77 ± 7.9 years, four men, four women; New York Heart Association functional class II/III, Table 1). Clinical data were collected with written informed consent after approval by the institutional review board. This was an observational study; therefore, the patients received standard therapy without any influence from the simulation results. The patients had follow-up echocardiograms three months after implantation and were classified as responders if the relative reduction in end-systolic volume (ESV) measured by the Simpson’s method ($\% \Delta {\rm{ESV}}\, = \,\left( {{\rm{ ES}}{{\rm{V}}_{{\rm{pre - CRT}}}}{\rm{ - ES}}{{\rm{V}}_{{\rm{post - CRT}}}}} \right){\rm{ }}/{\rm{ ES}}{{\rm{V}}_{{\rm{pre - CRT}}}}$ ) exceeded 15%. This definition of a responder was devised prior to this study.

**Table 1:** Patient characteristics

Pt #	Age	Gender	NYHA	Diagnosis
1	63	F	II	Sarcoidosis, complete AV-block
2	73	F	II	Hypertensive heart disease, complete AV-block
3	81	M	II	OMI, CLBBB
4	71	M	III	OMI, CLBBB
5	79	F	III	Aortic stenosis, CLBBB
6	79	M	III	DCM, CRBBB + left anterior fascicular block
7	81	M	II	Sarcoidosis, LBBB
8	89	M	III	Aortic regurgitation, complete AV-block

*OMI* old myocardial infarction, *CLBBB* complete left bundle branch block, *DCM* dilated cardiomyopathy, *CRBBB* complete right bundle branch block, *LBBB* left bundle branch block

### Simulation

Simulations were performed based on the data recorded before implantation. Researchers participating in the simulations were blinded to the outcome until the simulations were completed. The details of the simulation method were described previously [19, 20] and are shown in the supplementary methods provided in Online Resource 1.

### Patient-specific heart model

The finite element method was used for simulation with 3D models of ventricles, and upper body (torso) constructed from multi-slice CT images was subdivided into finite elements. To each of these elements, molecular models of cardiac electrophysiology with a spatially detailed sarcomere model [21] representing endocardial, midmyocardial (M-), or epicardial cells [22] were implemented in appropriate locations [17, 18]. The propagation of excitation was simulated by solving the bidomain equations. To reproduce the anisotropy in electrical conduction and mechanical response, we mapped the fiber orientation using the rule-based method [23]. The Purkinje fiber network was modeled as a thin layer on the endocardial surface with higher conduction velocity, and, in the case of left bundle branch block (LBBB), ventricular activation was started only on the right ventricular side. The infarcted region was determined from an echocardiogram and ECG. Initially, the inactive fibrous tissue property was assigned to akinetic segments as shown by an echocardiogram. The boundary of this region was then modified iteratively until the simulated ECG matched the clinically measured ECG. For cases of sarcoidosis, we changed the contractility and conduction velocity of the ventricle homogeneously because no focal abnormalities were reported by an echocardiogram.

**Fig. 1:**
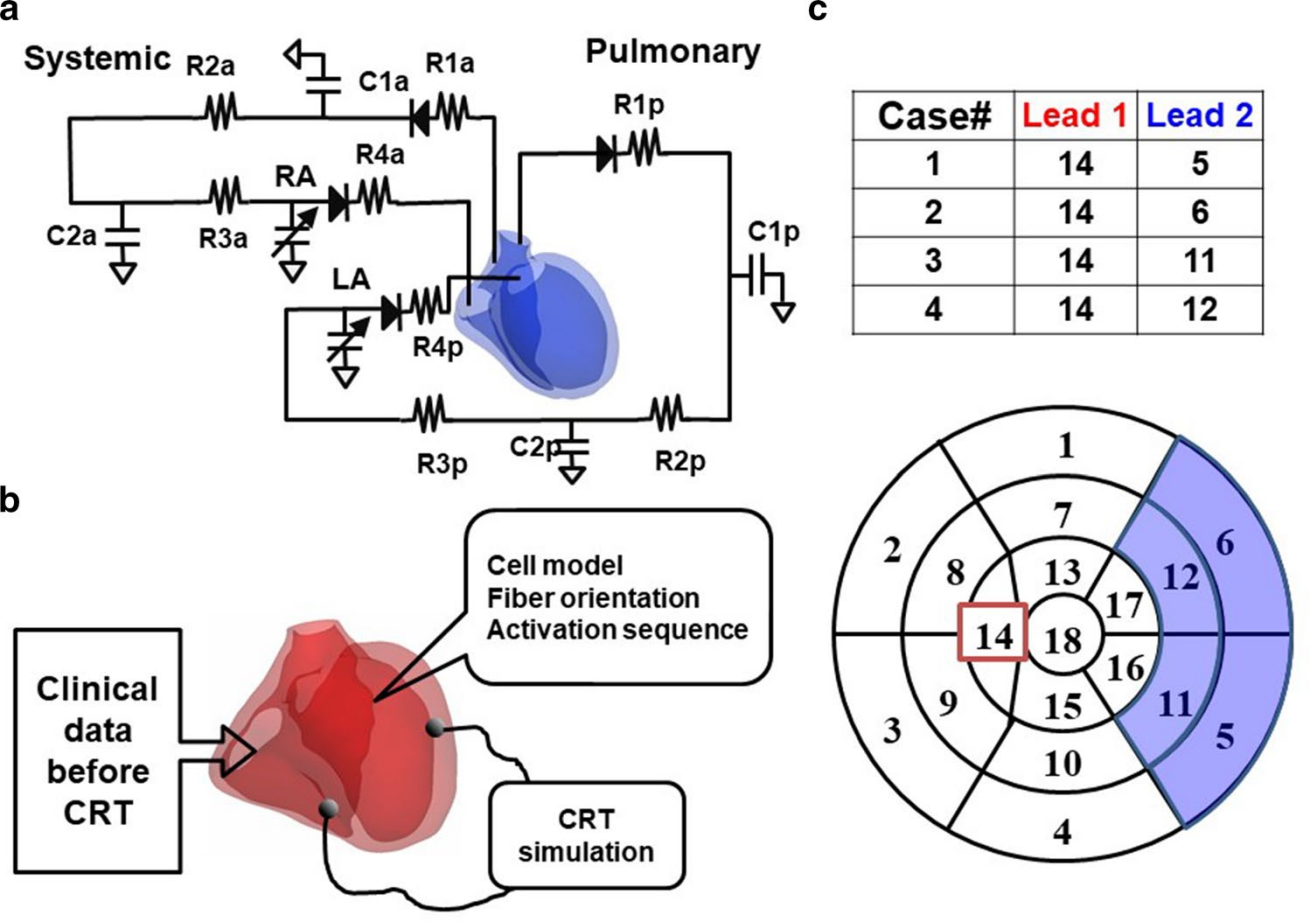
CRT simulation. **a** Electrical circuit analog of systemic and pulmonary circulations. C*; capacitance R*; resistance **b** A diagram of CRT simulation **c** Lead positions. Pacing leads were placed in one of four combinations of segments indicated in the bull’s eye view of the left ventricle

To save computational time and cost, we modeled only ventricles, and the time-varying elastance models of atria were connected with an electrical circuit analog of circulation (Fig. 1a), the parameter values of which were adjusted for each patient [19, 20, 24] (see supplementary Table 2 in Online Resource 1). Finally, contractility of the ventricles was adjusted to match the EF and non-invasive arterial blood pressure of each patient. Because invasive hemodynamic study was not available for any of these patients, end-diastolic pressure values were assigned depending on the functional class according to the literature [25].

### CRT simulation

For each heart model, we performed CRT simulation without changing any parameters of the heart, torso, and circulation determined for the model before the treatment. Simulation was performed with the four most commonly used lead positions in each patient model. One of the pacing leads was fixed in the right ventricular apex and the other was placed in either the basal or mid-portion of the anterolateral or posterolateral segment (Fig. 1b, c). VV delay was set at 0 ms. From the simulation results, we calculated both electrophysiological and mechanical parameters. The first-order derivative of the left ventricular pressure (dP/dt) was calculated by the numerical differentiation of simulated left ventricular pressure [26].

### Computation

All program codes were written in-house. They have been registered as intellectual property of the University of Tokyo. The computational time for a single beat was about 50 min for electrophysiological simulations and 30 min for mechanical simulations using 127 cores.

### Statistics

Data are presented as average ± standard error of mean (SEM).

## Results

### Clinical results

**Fig. 2:**
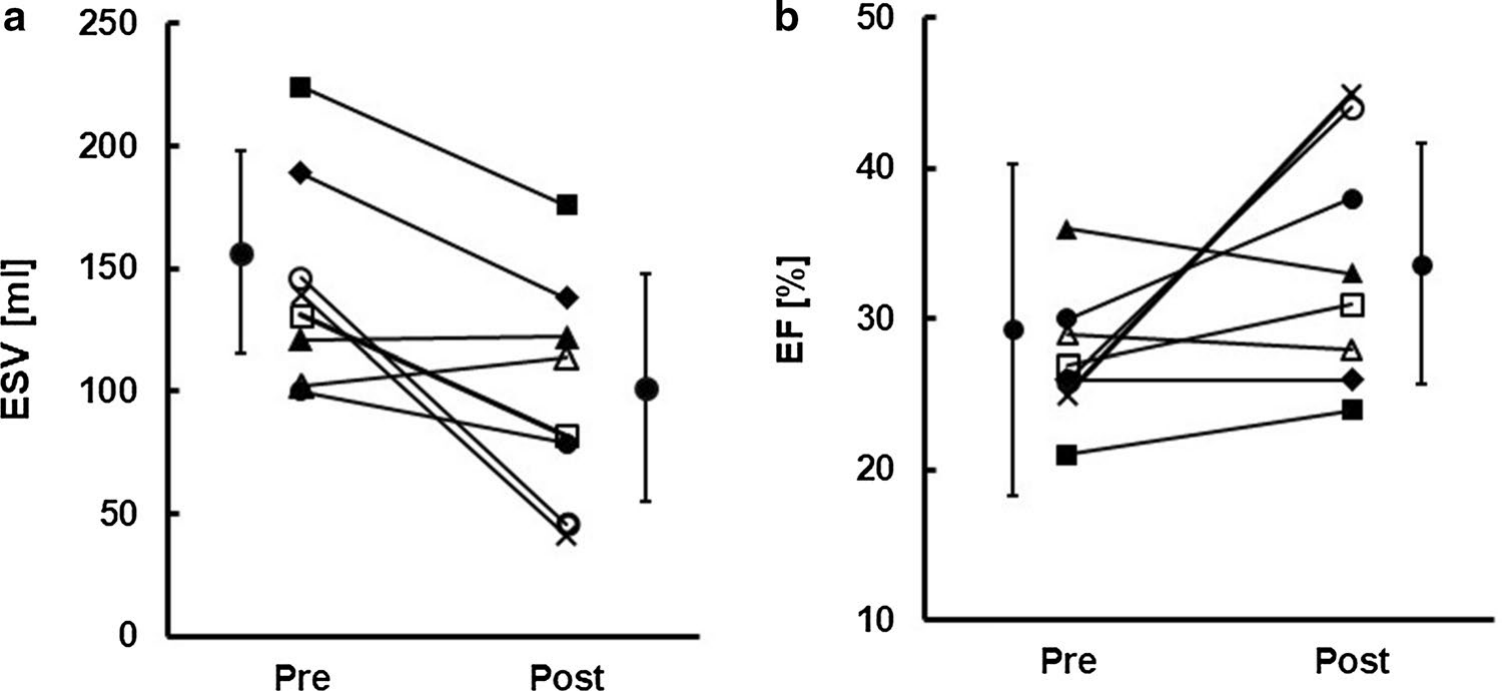
Clinical effect of CRT on ESV and the EF.** a** Comparison of ESV between pre- and post-CRT **b** Comparison of the EF between pre- and post-CRT. In both graphs, the same symbols are used for each patient. Mean and SEM are indicated beside the symbols

In the echocardiograms taken at the three-month follow-up, significant reductions of ESV (> 15%) were observed in six patients (responders, patient #s 1, 2, 3, 4, 5, and 8) (Fig. 2a). Patient #s 6 and 7 were judged as non-responders. We also noted that higher degrees of recovery exceeding 65% were introduced in two patients (super-responders, patient #s 2 and 8) (x and open circle in Fig. 2a, b). EF improved in five of the responders and increased by more than 15% in the two super-responders. In the remaining responders, despite the significant reduction of ESV, EF did not change at all (closed diamond in Fig. 2), suggesting that EF is not a reliable biomarker for the evaluation of CRT effect.

### CRT simulation

**Fig. 3:**
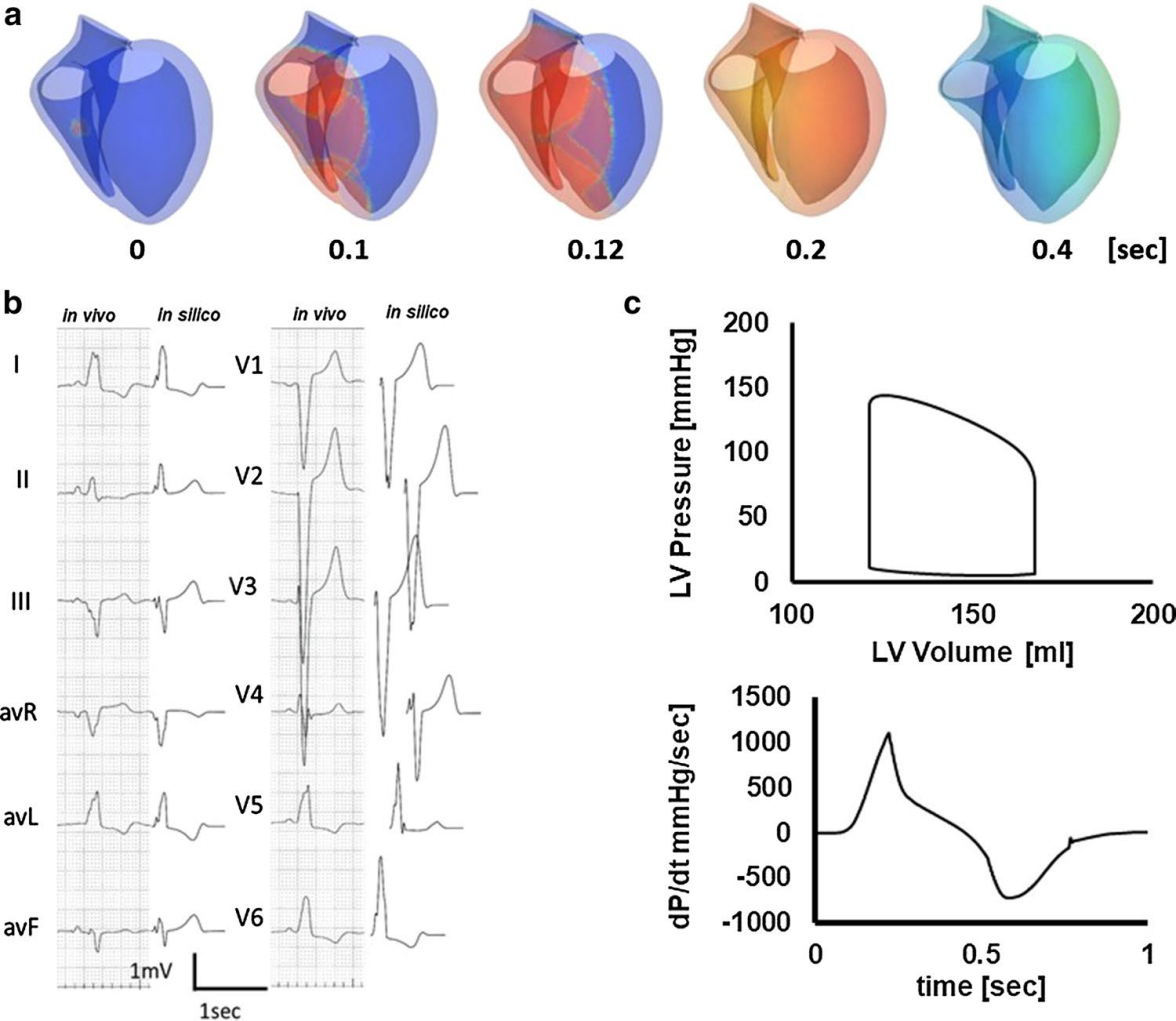
Patient-specific model of the failing heart. **a** Time-lapse images showing propagation of activation (color) and contraction. The numbers indicate the time after the onset of activation. **b** Actual (left column) and simulated (right column) ECG **c** Pressure–volume loop of the left ventricle (upper panel) and dP/dt (lower panel)

**Fig. 4:**
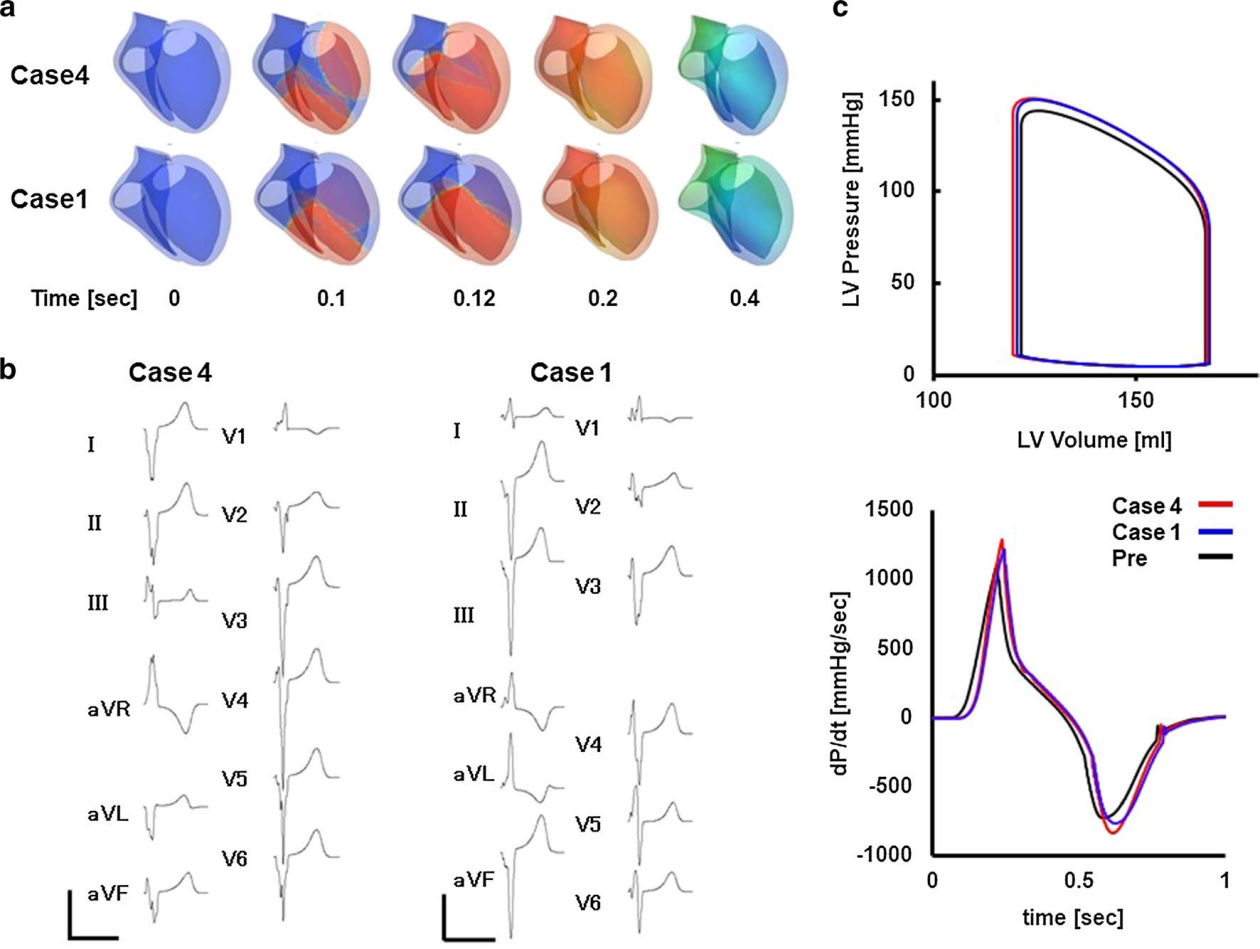
CRT simulations. **a** Propagation of activation and contraction under CRT that produced the best outcome (case 4) and the worst outcome (case 1). **b** Simulated ECGs of case 4 (left) and case 1 (right). **c** Comparisons of pressure–volume loops (upper panel) and dP/dt (lower panel) among pre-CRT, case 4 and case 1

Figure 3 shows an example of a patient-specific model of a failing heart with conduction block (patient #5). Ventricular activation propagates slowly from the right side of the ventricular septum spreading to the left ventricle (Fig. 3a, animation in Online Resource 3). With this activation sequence, the surface ECG of this patient was successfully reproduced (Fig. 3b). Figure 3c shows the left ventricular pressure–volume relationship and dP/dt before CRT (animation in Online Resource 4). With this heart model, we performed CRT simulations of four patterns of lead positions and identified the best (case 4, animation in Online Resource 5) and the worst (case 1, animation in Online Resource 6) lead positions (patient #5 in Table 2). Compared with case 1, activation covers the whole ventricle faster and QRS complexes were narrower in case 4 (Fig. 4a, b). Left ventricular pressure, stroke volume, and maximum d*P*/d*t* (d*P*/d*t*_max_) were all greater with case 4 (Fig. 4c).

**Table 2:** Simulated CRT effect

Pt #	Lead position	EF [%]	LVESV [ml]	LVEDV [ml]	d*P*/d*t*_max_ [mmHg/s]	Total activation time [msec]
1	Pre	20.4	256	322	1001	182
	1	22.8	250	324	1127	136
	2	22.9	250	325	1145	97
	3	23	248	322	1143	118
	4	23.3	248	323	1169	157
2	Pre	24	138	182	852	182
	1	24.9	137	183	951	134
	2	24.9	137	183	953	116
	3	25.1	137	183	958	136
	4	25	137	183	952	135
3	Pre	26	150	202	1181	227
	1	26	151	203	1237	174
	2	27	145	199	1345	184
	3	27	146	199	1335	185
	4	30	135	193	1508	156
4	Pre	30	153	219	1348	164
	1	31.8	139	204	1506	115
	2	31.7	143	209	1494	147
	3	32.5	137	203	1568	127
	4	32.7	139	207	1571	135
5	Pre	27.1	123	168	1126	175
	1	28.3	121	169	1249	136
	2	28.3	121	169	1301	115
	3	28.4	121	169	1268	155
	4	28.6	120	169	1321	115
6	Pre	35.8	158	245	1008	127
	1	36.3	157	246	977	126
	2	33.8	167	253	923	114
	3	36.2	157	246	980	95
	4	34.1	166	252	924	126
7	Pre	28.9	129	182	700	135
	1	29.9	126	180	768	115
	2	29.6	127	181	768	95
	3	30	126	180	772	115
	4	29.7	127	180	776	99
8	Pre	25	139	185	550	203
	1	26.1	137	185	621	173
	2	26.3	137	186	662	155
	3	26.2	136	185	626	177
	4	26.8	136	186	676	153

**Fig. 5:**
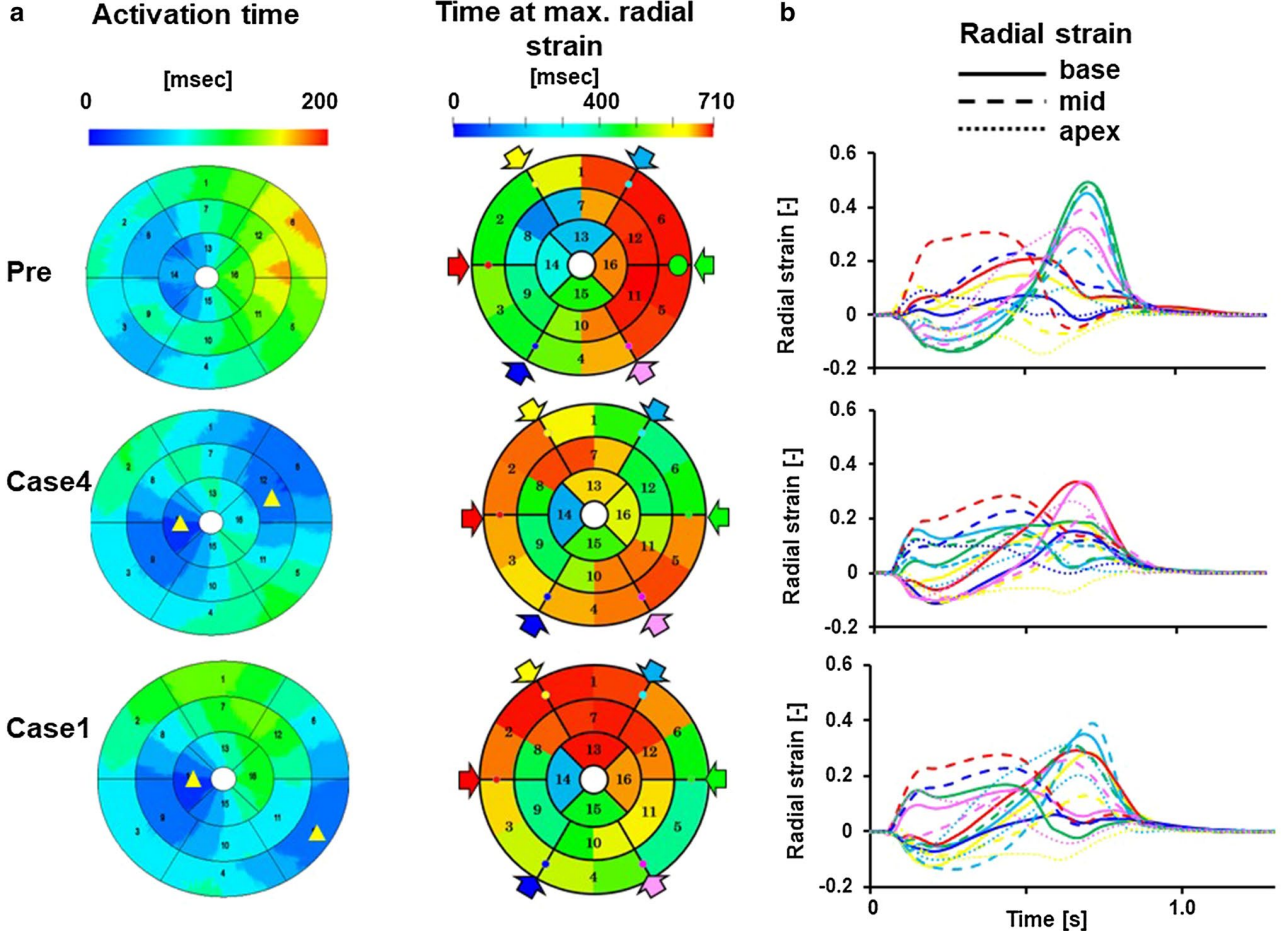
Evaluation of dyssynchrony. **a** Electrical activation time and time at maximum radial strain are shown by color in the bull’s eye view of the left ventricle and compared among pre-CRT, case 4 and case 1. Yellow triangles indicate the approximate position of pacing sites. **b** Temporal changes in radial strain, which was measured in 16 segments, compared among pre-CRT, case 4, and case 1. Data were sampled at the base (solid lines), mid-ventricular (broken lines), and apical (dotted lines) levels in six circumferential locations, which are indicated by color arrows in the bull’s eye view. However, red and green were omitted at the apical level

Figure 5a compares the segmental variations in electrical activation time, time at the maximal strain, and the temporal changes in radial strain in the basal segments before and after CRT. Before CRT (Pre), a significant delay in activation was observed in the anterolateral segments, which disappeared by the bi-ventricular pacing. The maximum values of activation time indicate the time required for the electrical activation to cover the whole ventricle (total activation time), which decreased from 175 ms (pre) to 136 ms (Case 1) and 115 ms (Case 4). Reflecting the delay in activation, time at the maximum radial strain was also delayed in the corresponding segment. Temporal changes in strain sampled at 16 segments are shown in Fig. 5b, where data were sampled at the base, mid-ventricular, and apical levels in 6 circumferential locations indicated by color arrows in the bull’s eye view. Before CRT, the basal septal segments (black, red, and blue solid lines) exhibited transient thickening in the pre-ejection period (septal flash) but became thin (negative strain) in the late systole. Bi-ventricular pacing corrected this abnormality, and, in case 4, no segments except for the apex showed negative strain in late systole. Clinically, this patient was judged as a responder (%$\Delta$ ESV =   −  37).

**Fig. 6:**
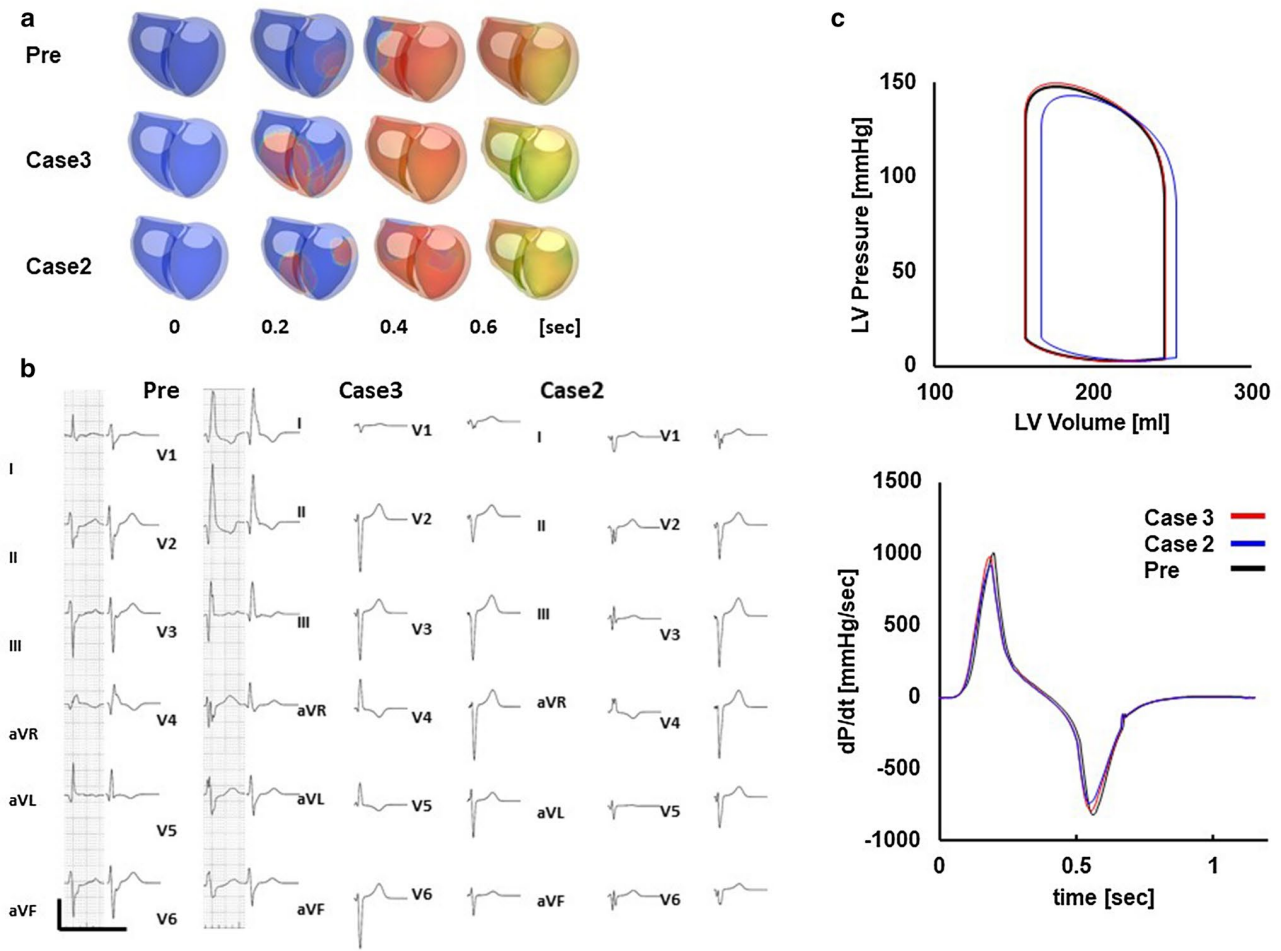
Patient-specific model and effect of CRT of a non-responder (patient #6). **a** Time-lapse images showing propagation of activation (color) and contraction before treatment (Pre), with the lead position that produced the best outcome (case 3) and the worst outcome (case 2). The numbers indicate the time after onset of activation. **b** Actual (left column) and simulated (right column) ECG under the three conditions. **c** Pressure–volume loop of the left ventricle (upper panel) and dP/dt (lower panel) under the three conditions (Pre: black line, case 3: red line, case 2: blue line)

**Fig. 7:**
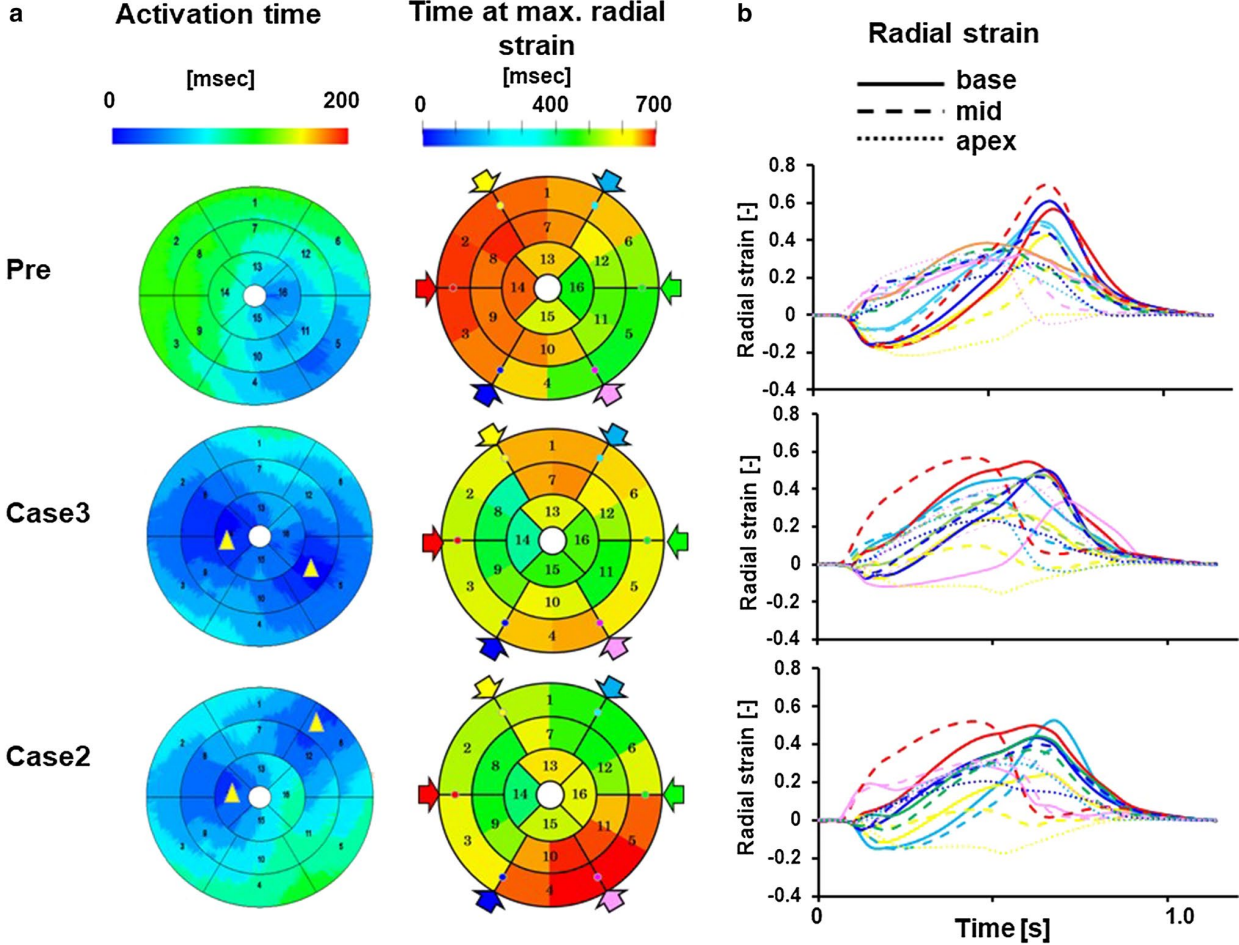
Evaluation of dyssynchrony for a non-responder (patient #6). **a** The activation time and the time at maximum radial strain are shown by color in the bull’s eye view of the left ventricle and were compared among pre-CRT, case 3 and case 2. Yellow triangles indicate the approximate position of pacing sites. **b** Temporal changes in radial strain, which were measured in 16 segments, were compared among pre-CRT, case 3 and case 2. Data were sampled at the base (solid lines), mid-ventricular (broken lines), and apical (dotted lines) levels in six circumferential locations, which are indicated by color arrows in the bull’s eye view. However, red and green were omitted at the apical level

Patient #6 was judged as being a non-responder (%$\Delta$ ESV = 0.8). Because this patient had right bundle branch block (RBBB) with left axis deviation (LAD) suggestive of left anterior fascicular block, activation propagated from the posterolateral wall and the basal septum was the last region to be activated in the left ventricle (Fig. 6a Pre, animation in Online Resource 7). With this activation sequence, ECG characteristics of typical RBBB and LAD that were observed in this patient were successfully reproduced (Fig. 6b Pre). Similarly to the previous case (patient #5), we performed CRT simulations of four patterns of lead positions and identified the best (case 3, animation in Online Resource 8) and the worst (case 2, animation in Online Resource 9) lead positions. In both lead positions, RBBB features of ECG disappeared (Fig. 6b, case 3 and 2), but contractile function did not appreciably change (Fig. 6c). As shown in Fig. 7a, the activation time was relatively short even before treatment (127 ms) and CRT (case 3) further shortened it to 95 ms. However, the wall motion abnormality persisted (Fig. 7b), which suggested that electrical delay was not an appropriate therapeutic target for this patient. The data for all patients are shown in Table 2 and the Supplementary figures in Online Resource 2.

### Prediction of non-responders

**Fig. 8:**
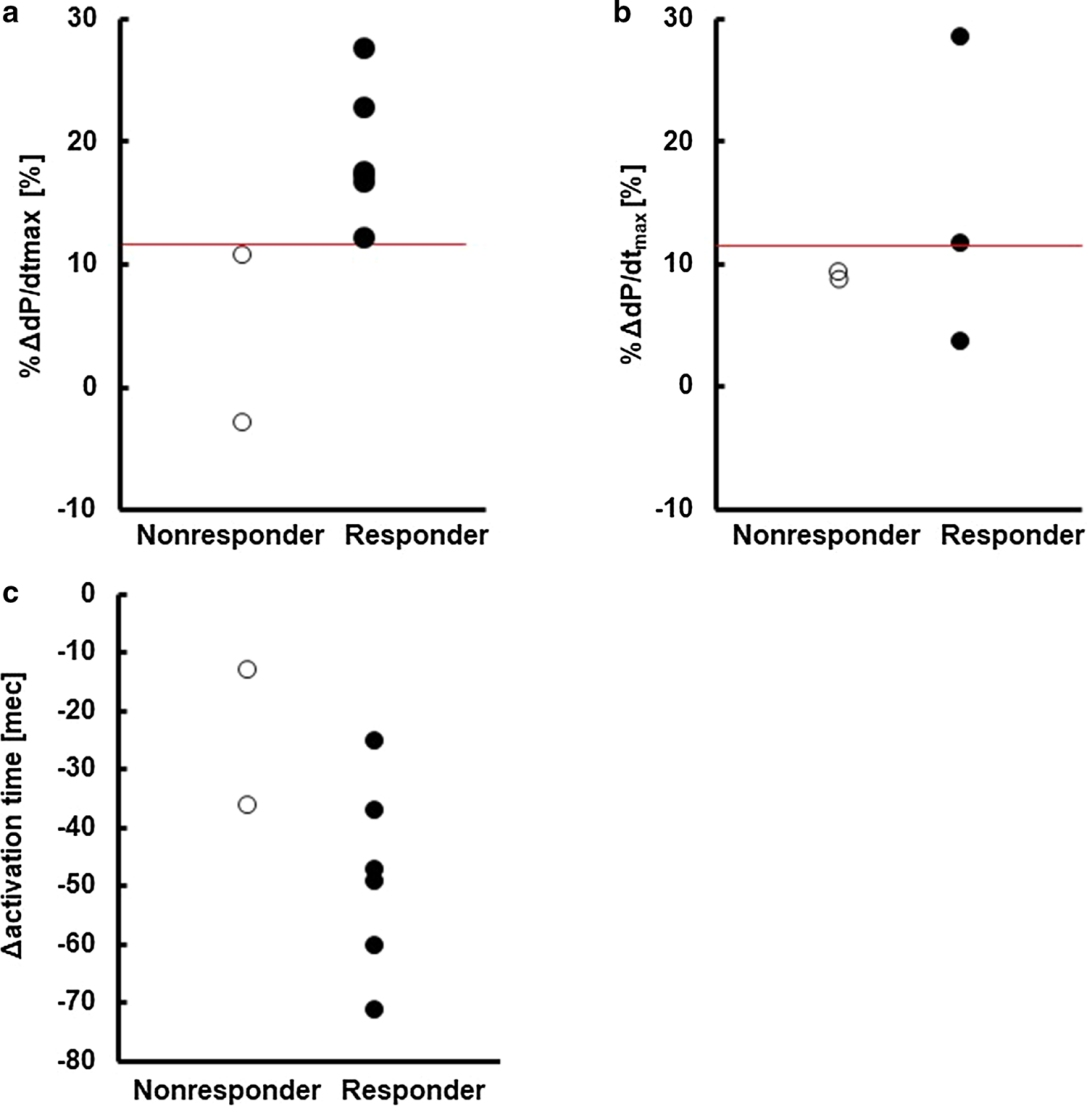
Prediction of non-responders. **a** Relative changes in d*P*/d*t*_max_ (%Δd*P*/d*t*_max_) of responders (closed circle) versus non-responders (open circle). The red line indicates the cutoff value. **b** Relative changes in d*P*/d*t*_max_ (%Δd*P*/d*t*_max_) of responders (closed circles) versus non-responders (open circles) in our previous study [19]. The red line indicates the cutoff value determined in panel (**a)**. **c** Relative changes in activation time (Δactivation time) of responders (closed circle) versus non-responders (open circle)

In our previous studies, a change in the maximum value of dP/dt (ΔdP/dt_max_) was identified as the sensitive marker of CRT effect [19, 20]. However, considering the variability in baseline dP/dt_max_ among the patients, we adopted the relative change in d*P*/d*t*_max_ (%Δd*P*/d*t*_max_) in this study and hypothesized that the patients for whom CRT fails to elicit a significant gain in %Δd*P*/d*t*_max_ even at the best lead position would be the non-responders. Figure 8a compares %Δd*P*/d*t*_max_ at the best lead position between the responders (closed circle) and the non-responders (open circle). Although the difference was small, simulated %Δd*P*/d*t*_max_ value could identify the responders. To further test the predictability of %Δd*P*/d*t*_max_, we selected five patients (see Supplementary Table 3 in Online Resource 1) from the study subjects in our previous study [19], of which follow-up echocardiograms at three months or later were available and plotted the data in a similar manner (Fig. 8b). In four out of five patients, we can correctly identify the responders and non-responders using the cutoff value obtained in Fig. 8a (red line: %Δd*P*/d*t*_max_ = 11.6). On the other hand, consistent with previous studies, decreases in total activation time by the pacing (Δ*d* activation time) did not predict the response to CRT (Fig. 8c) [19, 27].

## Discussion

CRT can be an effective therapy for end-stage heart failure [4], but identifying the significant number of non-responders remains a serious problem with this approach. While clinical studies continue to seek biomarkers that accurately identify the responders to CRT, simulation studies have also contributed to solving this problem. To date, most of these studies have attempted to understand the mechanisms of CRT [28–30], but we can also find reports reproducing the patient-specific pathology and responses to CRT [12, 31, 32]. Among these, Okada et al. applied CRT simulations to nine patient-specific heart models created based only on the data recorded before pacemaker implantation. They obtained a significant correlation between Δd*P*/d*t*_max_ by simulation and improvement in EF observed clinically [19]. In that retrospective study, however, they simulated the bi-ventricular pacing with the actual lead position, which we cannot know before the treatment.

In the current study, aiming at the clinical application of CRT simulation for the identification of non-responders, we avoided the use of information after the implantation and tested four patterns of commonly used lead positions. Based on our hypothesis that “if even the best lead position does not produce a significant therapeutic effect, the patient will be a non-responder,” we compared the greatest %Δd*P*/d*t*_max_ and the clinical outcome for each patient. Although the number of study subjects was small, we could identify non-responders.

**Fig. 9:**
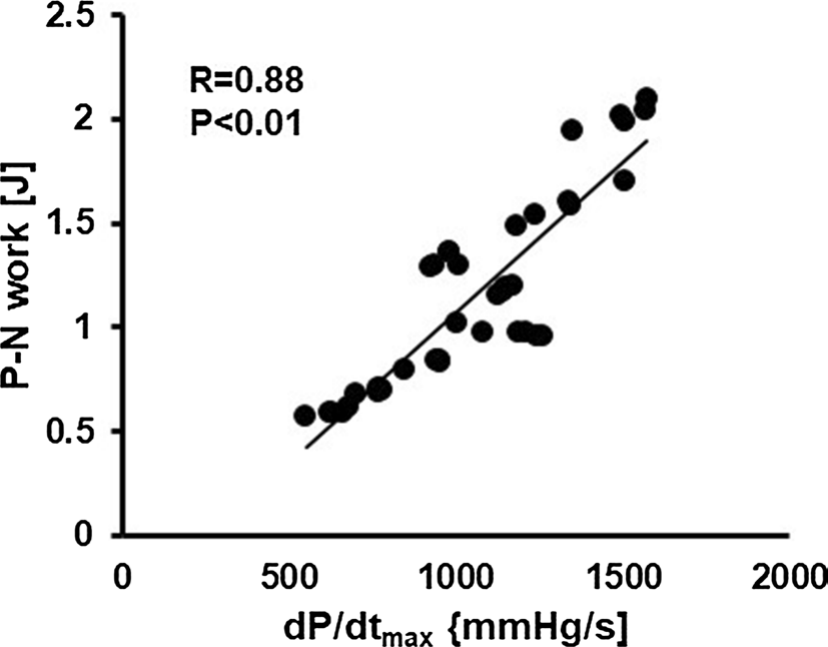
Dyssynchrony and ventricular pressure development. The relationship between the net positive work (P − N work) is plotted against d*P*/d*t*_max_ for all the lead positions in all of the study subjects. A linear regression line applied between them is also shown

Various biomarkers are currently used for the criteria of responders, such as functional class, six-minute walk, EF, and end-diastolic or -systolic left ventricular volume [33]. From these, we adopted the reduction in ESV at three months after the implantation, which is an index of reverse remodeling associated with a better prognosis of the patients [34]. However, the question remains for why the simulated acute improvement in d*P*/d*t*_max_ can predict the ventricular reverse remodeling in the chronic phase. An abnormal stretch of the ventricular wall triggers the pathological hypertrophy and dilatation of ventricle, and various interventions with unloading effects have been reported to induce reverse remodeling [35, 36]. In the dyssynchronous heart, the early activated segment pulls the delayed activated segment to cause abnormal stretch, which can be mitigated by CRT. From the viewpoint of ventricular mechanics, the stretched segment absorbs the work (negative work: N) done by the contracting segment (positive work: P), thereby impairing the pressure development. We calculated the effective work of the left ventricle during systole by subtracting the negative work from the positive work (P–N work). Work was calculated as the product of stress and strain in each element and summed for the entire left ventricle. We, then, plotted the P–N work against the d*P*/d*t*_max_ as an index of pressure development. Figure 9 summarizes all the data simulated for all lead positions for all patients. A significant correlation between d*P*/d*t*_max_ and P–N work suggests that dP/dt_max_ can be an indicator of abnormal stretch which promotes the pathological remodeling.

**Fig. 10:**
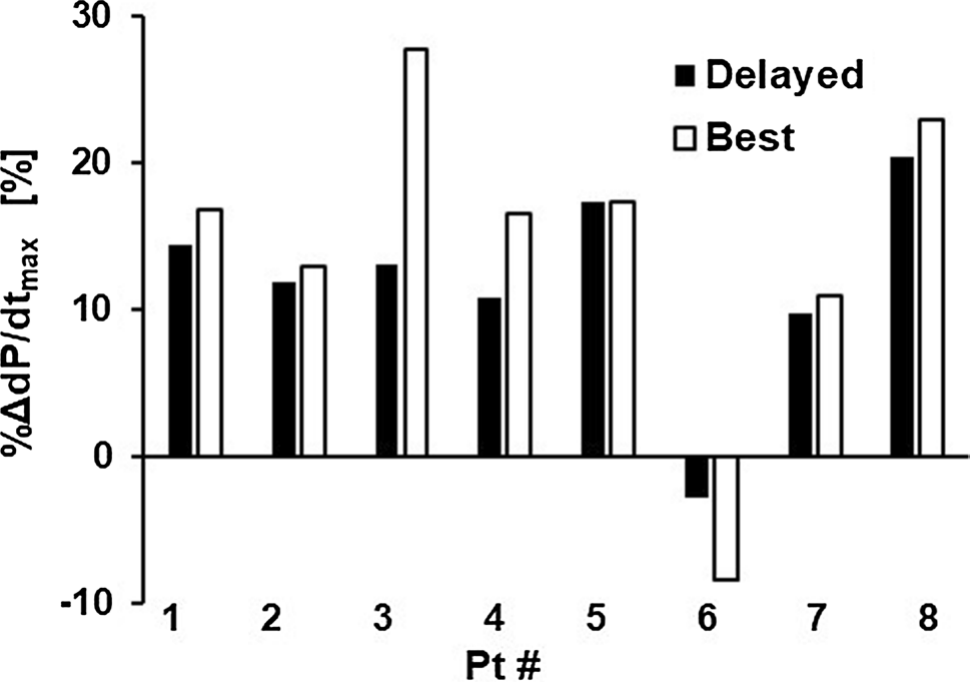
Effect of the lead position. Relative improvements in d*P*/d*t*_max_ (%d*P*/d*t*_max_) were compared between the lead positions in the most delayed area (black bars) and those with the best result (white bars) for each patient

Prolongation of the QRS duration is the class I recommendation for CRT in the guidelines [37]. Therefore, correction of electrical delay by placing the left ventricular lead in the late-activated area is a reasonable strategy for maximizing the effect of CRT. However, in patient #6, shortening of the QRS duration did not improve pumping function. Furthermore, when we examined the improvement of %d*P*/d*t*_max_ by placement of the left ventricular lead in the late-activated area in each subject, the largest improvement was achieved in only two of eight patients (Fig. 10). As discussed by Kass [38], multiple factors, including heterogeneity in wall geometry/size, contractile function, and cellular conduction, could decouple electrical delay and mechanical dyssynchrony. Therefore, placement of the lead in the most delayed area is not always the best strategy for achieving optimal CRT. A heart simulator coupling electrical and mechanical activities is a useful tool not only for predicting the effect of CRT but also for suggesting the optimal lead position for the CRT candidate.

The significance of biomarkers can also be evaluated using simulation results. To date, while multi-center trials have failed to show the usefulness of echo-dyssynchrony parameters for predicting the response of CRT, observational studies have shown that the presence of septal flash is a robust predictor of responders [39, 40]. In fact, we clearly observed the presence of septal flash and its disappearance by CRT in one of the responders (patient #5). A similar observation was made only in three of six responders. Septal flash mainly reflects the activation delay in the septum, which is the target of CRT. However, in addition to the lack of a gold standard for the assessment of septal flash, several conditions, such as regional loading and/or contractile abnormality, could obscure the appearance of septal flash even with left bundle branch block [41]. Further studies are required to fully examine the mechanisms and usefulness of septal flash as a predictor of the response to CRT.

Overall, the current study produced a promising result, which provokes a need for future study including a larger number of subjects. Nevertheless, this study has several limitations. Firstly, to save the computational cost, we fixed the VV and AV delays, both of which are known to have significant impacts on the CRT effect and thus should be included. Secondly, while responders were judged based on the reduction of ESV three months after implantation (chronic effect), the CRT simulation was applied to the heart models and vascular parameters before the treatment (acute effect), based on the assumption that the acute unloading effect by CRT would lead to the favorable remodeling in the remote phase, thus ignoring the remodeling processes at the cellular and tissue levels. The remodeling process and mechanisms at the cellular and tissue levels [42] should be considered in future modeling, but individualized data at these levels are currently hard to obtain. Finally, utilization of data not included in the current simulation would potentially improve the predictive ability of simulation. For instance, viability of the myocardium in patients with myocardial infarction and cardiac sarcoidosis was evaluated by an ECG and echocardiogram because advanced imaging data, such as gadolinium-enhanced magnetic resonance imaging were not available for the study subjects. However, if the feasibility of prediction without such expensive modalities is confirmed in a future study, this would be beneficial for patients. Furthermore, when we applied the newly determined cutoff value to the data from previous publication, one responder was judged as a non-responder (Fig. 8b). The diagnosis of this patient was cardiac sarcoidosis. Steroid therapy, when started simultaneously with CRT, might have altered the clinical course in this patient. Recently, machine learning using the patients’ baseline characteristics of the patients has been applied to the prediction of response to CRT, but the results are not necessarily very remarkable to date [43, 44]. A composite evaluation of machine learning scores and simulation results would potentiate the power of these two approaches.

## Conclusions

We performed CRT simulations with individualized heart models created using data obtained only before implantations for eight patients with heart failure. Simulated changes in dP/dt_max_ could successfully identify the non-responders to CRT.

## Electronic supplementary material

Below is the link to the electronic supplementary material.

Supplementary file1 (PDF 382 kb)

Supplementary file2 (PDF 5414 kb)

Supplementary file3 (MP4 8030 kb)

Supplementary file4 (MP4 195 kb)

Supplementary file5 (MP4 203 kb)

Supplementary file6 (MP4 210 kb)

Supplementary file7 (MP4 229 kb)

Supplementary file8 (MP4 190 kb)

Supplementary file9 (MP4 192 kb)
