# Supplementary material for: Patient-specific heart simulation can identify non-responders to cardiac resynchronization therapy
Source: Heart Vessels. 2020 Mar 12;35(8):1135–47. doi: 10.1007/s00380-020-01577-1 (PMC7332486; doi:10.1007/s00380-020-01577-1)
Supplement: Supplementary file 1 — Supplementary file1 (PDF 382 kb) [file 380_2020_1577_MOESM1_ESM.pdf]

Supplementary materials 1

Supplementary methods

1 Heart model

1.1 Mesh generation:

Segmentations of the multi-detector CT scan data taken at end-diastole were performed using commercial software (VirtualPlace; AZE Ltd., Tokyo, Japan). Based on these data, finite element meshes of the ventricles and torso were created. We used voxel meshes of the heart (size 0.2mm) and torso (size 1.6m) for electrophysiology and a tetrahedral mesh of the ventricles (size 2mm) for mechanical analyses. To these heart models, fiber orientations were mapped using the rule-based method[1].

1.2 Electrophysiology:

We solved the following bidomain equations

$$\beta\left(C_m\frac{\partial V}{\partial t}+I_{ion}\right)=I_{stim}-\frac{\partial}{\partial x_i}\left(G_{ij}^E\frac{\partial \emptyset^E}{\partial x_i}\right) \qquad (1)$$

$$\beta\left(C_m\frac{\partial V}{\partial t}+I_{ion}\right)=-I_{stim}-\frac{\partial}{\partial x_i}\left(G_{ij}^I\frac{\partial \emptyset^I}{\partial x_i}\right) \qquad (2)$$

where $\emptyset^E$ and $\emptyset^I$ are the extra- and intra cellular potential respectively, V = $\emptyset^I-\emptyset^E$ is transmembrane voltage, β is the surface-to-volume ratio of the tissue, Cm is membrane capacitance, t is time, G_{ij}^E and G_{ij}^I are the intra- and extracellular anisotropic conductivity

tensors, x_i and x_j are the tensor notation of the x, y, and z coordinates, and I_{ion} is the sum of ionic transmembrane currents calculated by the human ventricular myocyte model of electrophysiology. Values of conductivity used for the heart and torso were adopted from literature [2-4] (supplementary Table 1). We used the human cell model of electrophysiology by tenTusscher et al. [5] for ventricular tissue. The Purkinje network was modeled as a thin layer with high conduction velocity on the endocardial surface [6-8].

1.3 Sarcomere model:

We used the spatially-detailed model of sarcomere dynamics we developed [9-11], in which 38 myosin heads on the thick filament and 32 tropoin/tropomyosin regulatory units on the thin filament in half sarcomere were modeled. Frank-Starling mechanism was realized by the overlap of thick and thin filaments. Co-operativity among the neighboring myosin heads was introduced for the transition rates between detached and attached states in the crossbridge cycling.

1.4 Mechanics:

The discretized form of equation of motion was solved in the tetrahedral mesh:

$$\nabla \cdot \boldsymbol{\sigma} + \boldsymbol{f} = 0 \tag{3}$$

where $\boldsymbol{\sigma}$ is the Cauchy stress tensor and $\boldsymbol{f}$ is the body force. $\boldsymbol{\sigma}$ can be described as:

$$\sigma = J^{-1}\boldsymbol{FSF}^{T}, \quad (4)$$

where $\boldsymbol{F} = \frac{\partial x}{\partial X}$ is the deformation gradient tensor, $\boldsymbol{S}$ is the second Piola-Kirchoff stress and $\boldsymbol{J}$ =det$\boldsymbol{F}$ is the Jacobian determinant.

S was given as:

$$\boldsymbol{S} = 2\frac{\partial W_{pass}}{\partial \boldsymbol{C}} - \mathrm{p}\boldsymbol{C}^{-1} + \boldsymbol{S}^{\boldsymbol{a}}, \quad (5)$$

where $\boldsymbol{C}$ is right Cauchy-Green deformation tensor and p is the hydrostatic pressure.

Strain energy function (W_{pass}) for the passive property was calculated as the sum of the two functions proposed by Humphrey et al. (W_{Hum}) [12] and Lin et al. (W_{Lin}). [13]

$$W_{pass} = \mathrm{a} \cdot W_{Hum} + b \cdot W_{Lin} \quad (6)$$

$$W_{Hum} = c_1(\alpha - 1)^2 + c_2(\alpha - 1)^3 + c_3(I_1 - 1) + c_4(I_1 - 1)(\alpha - 1) + c_5(\alpha - 1)^2 \quad (7)$$

$$W_{Lin} = c_6(e^Q - 1) \quad (8)$$

$$\mathrm{Q} = c_7(I_1 - 3)^2 + c_8(I_1 - 3)(I_4 - 1) + c_9(I_4 - 1)^2 \quad (9)$$

$I_1 = trC$, $I_4 = \boldsymbol{NCN}$, $\alpha = \sqrt{\boldsymbol{NCN}}$, $\boldsymbol{C}$ is the right Cauchy-Green deformation tensor and $\boldsymbol{N}$ is the unit vector defining the preferred direction of muscle in the undeformed state. Parameter values of c_1=3080pa, c_2=3240pa, c_3=359pa, c_4=-1940pa, c_5=1660pa, c_6=82.3pa, c_7=7.3, c_8=1.86 and c_9=0.0640 were used, and a and b were adjusted to reproduce the diastolic pressure-volume relation estimated by the formula by Klotz et al. [14] in patients.

The active stress tensor $\boldsymbol{S}^{a}$ was calculated as:

$$S_a = \begin{bmatrix} S_{ff}^a & 0 & 0 \\ 0 & S_{ss}^a & 0 \\ 0 & 0 & S_{nn}^a \end{bmatrix}, \quad (10)$$

where *f*, *s*, and *n* indicate fiber direction, sheet direction, and sheet normal direction, respectively. S_{ss}^a=0, S_{nn}^a=0 and S_{ff}^a was the active force calculated by the above sarcomere model.

2. Circulatory model:

The finite element model of ventricles was connected to a lumped parameter model of systemic and pulmonary circulation (Fig. 1A) similar to Kerckhoffs et al. [15] and solved by the following equations.

Systemic circulation:

$$\mathrm{Pas=Vas/C1a} \quad (11)$$

$$\mathrm{Pvs=Vvs/C2a} \quad (12)$$

$$\mathrm{Q_{ao}} = \begin{cases} \frac{\mathrm{P_{LV}-P_{as}}}{\mathrm{R1a}} & (\mathrm{P_{LV}} > \mathrm{P_{as}}) \\ 0 & (\mathrm{P_{LV}} < \mathrm{P_{as}}) \end{cases} \quad (13)$$

$$\mathrm{Q_{as}} = (\mathrm{Pas} - \mathrm{Pvs})/\mathrm{R2a} \quad (14)$$

$$\mathrm{Q_{vs}} = (\mathrm{Pvs} - \mathrm{P_{RA}})/\mathrm{R3a} \quad (15)$$

$$\mathrm{Q_{mitral}} = \begin{cases} \frac{\mathrm{P_{LA}-P_{LV}}}{\mathrm{R4a}} & (\mathrm{P_{LA}} > \mathrm{P_{LV}}) \\ 0 & (\mathrm{P_{LA}} < \mathrm{P_{LV}}) \end{cases} \quad (16)$$

$$\frac{\mathrm{dV_{LA}}}{\mathrm{dt}} = \mathrm{Q_{VP}} - \mathrm{Q_{mitral}} \quad (17)$$

$$\frac{\mathrm{dV_{LV}}}{\mathrm{dt}} = \mathrm{Q_{mitral}} - \mathrm{Q_{ao}} \quad (18)$$

$$\frac{dV_{as}}{dt} = Q_{ao} - Q_{as} \quad (17)$$

$$\frac{dV_{vs}}{dt} = Q_{as} - Q_{vs} \quad (18)$$

Pas is the pressure of the systemic arteries, Pvs is pressure of the systemic veins, P_{LV} is pressure in the left ventricle, P_{LA} is pressure in the left atrium, Vas is volume of the systemic arteries, Vvs is volume of the systemic veins, V_{LV} is volume of the left ventricle, V_{LA} is volume of the left atrium, Q_{mitral} is mitral flow, Q_{VP} is pulmonary venous flow, Qao is aortic flow, Qas is flow out of the systemic arteries, and Qvs is flow out of the systemic veins.

Pulmonary circulation:

$$Pap = Vap/C1p \quad (19)$$

$$Pvp = Vvp/C2p \quad (20)$$

$$Q_{pa} = \begin{cases} \frac{P_{RV} - P_{ap}}{R1p} & (P_{RV} > P_{ap}) \\ 0 & (P_{RV} < P_{ap}) \end{cases} \quad (21)$$

$$Q_{ap} = (Pap - Pvp)/R2p \quad (22)$$

$$Q_{vp} = (Pvp - P_{LA})/R3p \quad (23)$$

$$Q_{tricus} = \begin{cases} \frac{P_{RA} - P_{RV}}{R4a} & (P_{RA} > P_{RV}) \\ 0 & (P_{RA} < P_{RV}) \end{cases} \quad (24)$$

$$\frac{dV_{RA}}{dt} = Q_{vs} - Q_{tricus} \quad (25)$$

$$\frac{dV_{RV}}{dt} = Q_{tricus} - Q_{pa} \quad (26)$$

$$\frac{dV_{ap}}{dt} = Q_{pa} - Q_{ap} \quad (27)$$

$$\frac{dV_{Vp}}{dt} = Q_{ap} - Q_{vp} \tag{28}$$

Pap is the pressure of the pulmonary arteries, Pvp is pressure of the pulmonary veins, P_{RV} is pressure in the right ventricle, P_{RA} is pressure in the right atrium, Vap is volume of the pulmonary arteries, Vvp is volume of the pulmonary veins, V_{RV} is volume of the right ventricle, V_{RA} is volume of the right atrium, Q_{tricus} is tricuspid flow, Q_{vs} is systemic venous flow, Qpa is pulmonary artery flow, Qap is flow out of the pulmonary arteries, and Qvp is flow out of the pulmonary veins. The right and left atria were modeled by the time-varying elastance model proposed by Kaye et al. [16].

3. Parameter personalization

3.1 ECG personalization:

We adjusted parameters to make a match between the simulated ECG and the actually recorded standard 12-lead ECGs of patients following the previously reported procedure [7]. First, we fitted the QRS complex by shifting standard endocardial earliest activation sites determined in our previous study[7]. In the case of left bundle branch block, endocardial earliest activation sites located in the right side of the heart were used. Next, the transmural distribution of different cell types, namely endocardial, M-, and epicardial cells, were adjusted to reproduce T-wave morphologies. Goodness of the fit was evaluated by visual inspection and the calculation of

cross-correlation (Rcc) between the simulated and actual ECGs.

$$R_{NCC} = \frac{\sum_{j=1}^{16}\sum_{i=1}^{N} A(i,j)\times B(i,j)}{\sqrt{\sum_{j=1}^{16}\sum_{i=1}^{N} A(i,j)^2\times \sum_{l=1}^{16}\sum_{k=1}^{N} B(k,l)^2}} \tag{29}$$

Rcc > 0.8 was taken as the sign of good agreement.

3.2 Personalization of systemic and pulmonary circulation:

We estimated initial values of vascular parameters based on ultrasonic cardiogram (UCG) recording, arterial blood pressure measurement and literature.

Stroke volume was calculated as the product of the end-diastolic volume derived from the finite element model and the ejection fraction by UCG. Cardiac output was then calculated by multiplying the stroke volume by heart rate. Mean arterial pressure (Pm) was estimated from systolic pressure (Ps) and diastolic pressure (Pd) by the following equation

$$\mathrm{Pm} = \frac{(Ps-Pd)}{3} + Pd, \tag{30}$$

and mean right atrial pressure was estimated from the diameter of inferior vena cava measured by UCG [17]. Total systemic resistance (=R1a+R2a+R3a) was calculated as (mean arterial pressure–mean right atrial pressure)/cardiac output. At this point we assigned 93% of total systemic resistance to R2a, 5% to R1a, and 2% to R3a [18-20]. Assuming that end-systolic pressure (Pes)

was equal to mean arterial pressure, we estimated the time constant of arterial pressure decay during diastole (τ) by fitting to the exponential function:

$$\mathrm{Pd} = \mathrm{Pes} \cdot e^{-\frac{t_d}{\tau}}, \quad (31)$$

where t_d is the diastolic time interval obtained from the UCG recording. [21] C1a was given by dividing the τ value by R2a. Guyton et al. estimated the ratio between venous and arterial capacitances at 30 in live dogs [22] while it was set at 70 in simulation of human heart failure by Kaye et al. [16]. We adopted the intermediate value of 40 in this study. Filling resistance of the tricuspid valve (R4a) was set at 0.0025mmHgs/ml [16].

Parameter values for the pulmonary circulation were determined in a similar way. 37%, 43% and 20% of the calculated total pulmonary resistance was assigned to R1p, R2p and R3p, respectively, and mitral filling resistance was set at 0.0025mmHg s/ml according to Kaye et al. [16]. Although pulmonary venous capacitance (C2p) and arterial capacitance (C3p) varied among studies, we adopted the value by Kaye et al. (C2p=7, C3p=4 ml/mmHg for 70kg body weight) and adjusted for the patient's weight. Stressed blood volume was set at 15% of total blood volume which was assumed to be 8% of body weight.

Similar to Kerckhoff et al. [15], we tuned the initial parameter values using a simpler model in which the finite element model of the ventricles in supplementary Fig. 1 was replaced with time-varying elastance models of the right and left ventricles [23]. Because this system runs

faster than real time, we could tune the parameter values efficiently. The program was written by LabVIEW 2012 (National Instruments, Austin, TX, USA) and the tuning was performed interactively starting from initial values.

Finally, parameter values thus determined were applied to the finite element model simulation. At this stage, contractility of the heart model was adjusted by multiplying adjusting factors to the active stress tensor $\boldsymbol{S}^a$.

Supplementary Table 1

Conductivities of the organs [mS/cm]	
Heart (intracellular)	
Fiber direction	6 .2
Fiber normal direction	2 .0
Heart (extracellular)	
Fiber direction	4 .0
Fiber normal direction	2 .0
Blood	7 .8
Atrium, Artery, Vein	7 .0
Muscle	2 .56
Lung	0 .83
Esophagus, Stomach, Bowel	2 .0
Spleen, Liver, Kidney	1 .67
Bone	0 .1
Fat	0 .5
Skin, Body Surface	80

Supplementary Table 2

Vascular parameters

Pt#	R1a [mmHg/ml/s]	R2a [mmHg/ml/s]	C1a [ml/mmHg]	R3a [mmHg/ml/s]	C2a [ml/mmHg]	R4a [mmHg/ml/s]
1	0.0359	0.682	2.26	0.016	3	0.0025
2	0.142	2.13	0.5	0.022	5.3	0.0025
3	0.072	1.5	0.5	0.0025	2.7	0.0025
4	0.011	2	0.76	0.01	8	0.0025
5	0.096	2.99	0.59	0.025	70	0.025
6	0.063	1.05	2	0.025	90	0.0025
7	0.078	1.45	1.11	0.027	111	0.0025
8	0.051	1.01	0.533	0.019	80	0.025

Pt#	R1p [mmHg/ml/s]	R2p [mmHg/ml/s]	C1p [ml/mmHg]	R3p [mmHg/ml/s]	C2p [ml/mmHg]	R4p [mmHg/ml/s]
1	0.009	0.024	27	0.0048	3.7	0.0025
2	0.04	0.11	14	0.036	5	0.0025

3	0.05	0.013	8	0.025	32	0.0025
4	0.018	0.048	12.7	0.01	8	0.0025
5	0.045	0.052	6.8	0.025	4	0.0025
6	0.02	0.06	11.4	0.025	2.5	0.0025
7	0.025	0.029	7.2	0.006	3.37	0.0025
8	0.011	0.08	3.8	0.058	2.2	0.001

Supplementary Table 3

Patient characteristics for ad hoc analysis [24]

Pt #	age	gender	NYHA	Diagnosis
1	60	M	II	Sarcoidosis, CRBBB, complete AV-block
2	61	F	II	Sarcoidosis, CLBBB, advanced AV-block
3	64	M	II	OMI, AF
4	73	M	-	DCM
5	48	F	II	DCM , CLBBB

CRBBB: complete right bundle branch block, CLBBB: complete left bundle branch block, DCM: dilated cardiomyopathy, OMI: old myocardial infarction, DCM: dilated cardiomyopathy

NYHA class was not evaluated for the patient 4 due to the gait disturbance.

Supplementary references

1. Hisada T, Kurokawa H, Oshida M, Yamamoto T, Washio T, Okada J-I, Watanabe H, Sugiura S, inventors; Japanese Science and Technology Agency, assignee. Modeling device, program, computer-readable recording medium, and method of establishing correspondence. USA patent US 8,095,321 B2. 2012 Jan. 10, 2012.

2. Camacho MA, Lehr JL, Eisenberg SR (1995) A three-dimensional finite element 40 model of human transthoracic defibrillation: paddle placement and size. IEEE Trans Biomed Eng 42:572-578.

3. Keldermann RH, ten Tusscher KH, Nash MP, Bradley CP, Hren R, Taggart P, Panfilov AV (2009) A computational study of mother rotor VF in the human ventricles. Am J Physiol 296:H370-379.

4. Panescu D, Webster JG, Tompkins WJ, Stratbucker RA (1995) Optimization of cardiac defibrillation by three-dimensional finite element modeling of the human thorax. IEEE Trans Biomed Eng 42:185-192.

5. ten Tusscher KHWJ, Noble D, Noble PJ, Panfilov AV (2004) A model for human ventricular tissue. Am J Physiol 286:H1573–H1589.

6. Okada J, Washio T, Maehara A, Momomura S, Sugiura S, Hisada T (2011) Transmural and apicobasal gradients in repolarization contribute to T-wave genesis in human surface ECG.

Am J Physiol 301:H200–H208.

7. Okada J, Sasaki T, Washio T, Yamashita H, Kariya T, Imai Y, Nakagawa M, Kadooka Y, Nagai R, Hisada T, Sugiura S (2013) Patient specific simulation of body surface ECG using the finite element method. Pacing Clin Electrophysiol 36:309-321.

8. Okada J-I, Yoshinaga T, Kurokawa J, Washio T, Furukawa T, Sawada K, Sugiura S, Hisada T (2015) Screening system for drug-induced arrhythmogenic risk combining a patch clamp and heart simulator. Sci Adv 1:e1400142.

9. Washio T, Okada J, Sugiura S, Hisada T (2011) Approximation for cooperative interactions of a spatially-detailed cardiac sarcomere model. Cell Mol Bioeng.

10. Sugiura S, Washio T, Hatano A, Okada J-I, Watanabe H, Hisada T (2012) Multi-scale simulations of cardiac electrophysiology and mechanics using the University of Tokyo heart simulator. Prog Biophys Mol Biol 110:380-389.

11. Panthee N, Okada J-I, Washio T, Mochizuki Y, Suzuki R, Koyama H, Ono M, Hisada T, Sugiura S (2016) Tailor-made heart simulation predicts the effect of cardiac resynchronization therapy in a canine model of heart failure. Med Image Anal 31:46-62.

12. Humphrey JD, Strumpf RK, Yin FCP (1990) Determination of a constitutive relation for passive myocardium: II. parameter estimation. J Biomech Eng 112:340-346.

13. Lin DHS, Yin FCP (1998) Constitutive law for mammalian left ventricular myocardium

in steady-state Barium contracture or tetanus. J Biomech Eng 120:504-517

14. Klotz S, Dickstein ML, Burkhoff D (2007) A computational method of prediction of the enddiastolic pressure-volume relationship by single beat. Nat Protoc 2:2152-2158.

15. Kerckhoffs RCP, Neal ML, Gu Q, Bassingthwaighte JB, Omens JH, McCulloch AD (2007) Coupling of a 3D finite element model of cardiac ventricular mechanics to lumped systems models of the systemic and pulmonic circulation. Annals of Biomedical Engineering 35:1-18.

16. Kaye D, Shah SJ, Borlaug BA, Gustafsson F, Komtebedde J, Kubo S, Magnin C, Maurer MS, Feldman T, Burkhoff D (2014) Effects of an internal shunt on rest and exercise hemodynamics: Results of a computer simulation in heart failure. J Cardiac Fail 20:212-221.

17. Rudski LG, Lai WW, Afilalo J, Hua L, Handschumacher MD, Chandrasekaran K, Solomon SD, Louie EK, Schiller NB (2010) Guidelines for the echocardiographic assessment of the right heart in adults: a report from the American Society of Echocardiography endorsed by the European Association of Echocardiography, a registered branch of the European Society of Cardiology, and the Canadian Society of Echocardiography. J Am Soc Echocardiogr 23:685-713.

18. Nichols WW, Pepine CJ, Geiser EA, Conti R (1980) Vascular load defined by the aortic input impedance spectrum. Fed Proc 39:196-201.

19. O'Rourke MF, Taylor MG (1967) Input impedance of the systemic circulation. Circ Res

20:365-380.

20. Westerhof N, Elzinga G, Sipkema P (1971) An artificial arterial system for pumping hearts. J Appl Physiol 36:123-127.

21. Sunagawa K, Maughan WL, Burkhoff D, Sagawa K (1983) Left ventricular inter- action with arterial load studies in isolated canine ventricle. Am J Phyisol 245:H773-780.

22. Guyton AC, Armstrong GG, Chipley PL (1956) Pressure-volume curves of the arterial and venous systems in live dogs. Am J Physiol 184:253-258.

23. Suga H, Sagawa K (1974) Instantaneous pressure-volume relationships and their ratio in the excised, supported canine left ventricle. Circ Res 35:117-126.

24. Okada J-I, Washio T, Nakagawa M, Watanabe M, Kadooka Y, Kariya T, Yamashita H, Yamada Y, Momomura S-I, Nagai R, Hisada T, Sugiura S (2017) Multi-scale, tailor-made heart simulation can predict the effect of cardiac resynchronization therapy. J Mol Cell Cardiol 108:17-23.
