# Supplementary material for: Patient-specific heart simulation can identify non-responders to cardiac resynchronization therapy
Source: Heart Vessels. 2020 Mar 12;35(8):1135–47. doi: 10.1007/s00380-020-01577-1 (PMC7332486; doi:10.1007/s00380-020-01577-1)
Supplement: Supplementary file 2 — Supplementary file2 (PDF 5414 kb) [file 380_2020_1577_MOESM2_ESM.pdf]

Supplementary materials 2

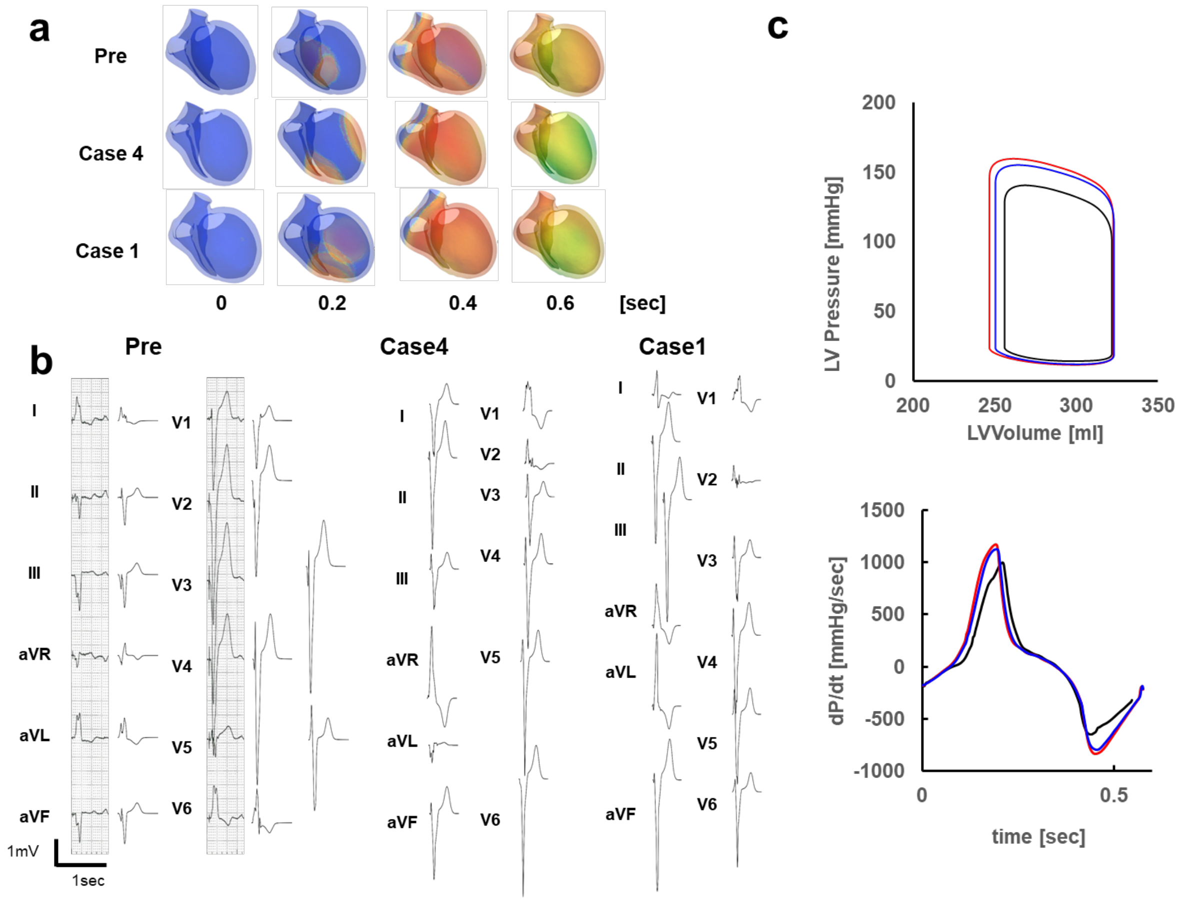

SFig.1 Patient-specific model and CRT effect in Patient #1

a: Time-lapse images showing the propagation of activation (color) and contraction before the treatment (Pre), with the lead position producing the best outcome (Case 4) and the worst outcome (Case 1). Numbers indicate the time after the onset of activation. b: Actual (left column) and simulated (right column) ECG under the three conditions. c: Pressure-volume loop of the left ventricle (upper panel) and dP/dt (lower panel) under the three conditions (Pre: black line, Case 4: red line, Case 1: blue line).

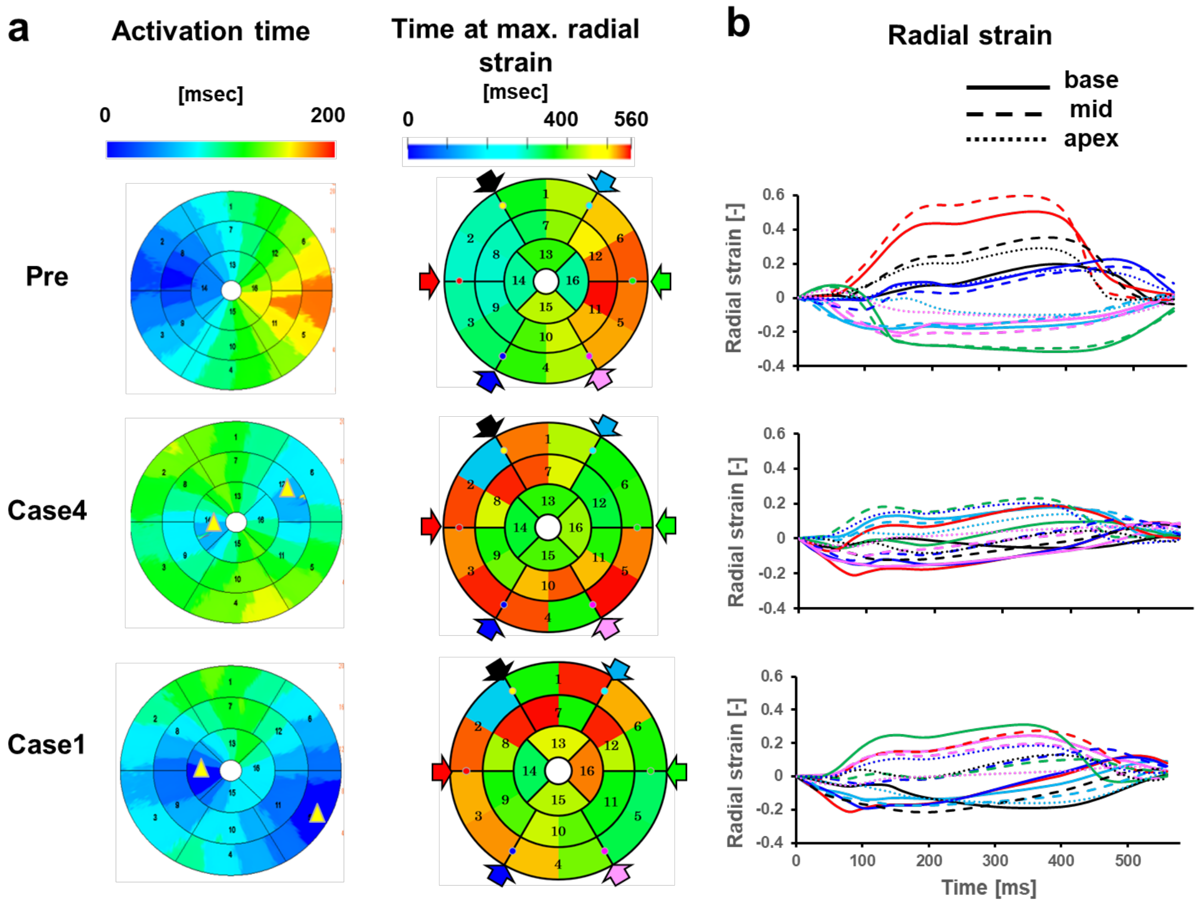

SFig.2 Evaluation of dyssynchrony

a: Activation time and time at maximum radial strain are shown by color in the bull's eye view of the left ventricle and compared among pre-CRT, case 4 and case 1. Yellow triangles indicate the approximate position of pacing sites. b: Temporal changes in radial strain, which was measured in 16 segments, compared among pre-CRT, case 4, and case 1. Data were sampled at the base (solid lines), mid-ventricular (broken lines) and apical (dotted lines) levels in six circumferential locations, which are indicated by color arrows in the bull's eye view. However, red and green were omitted at the apical level.

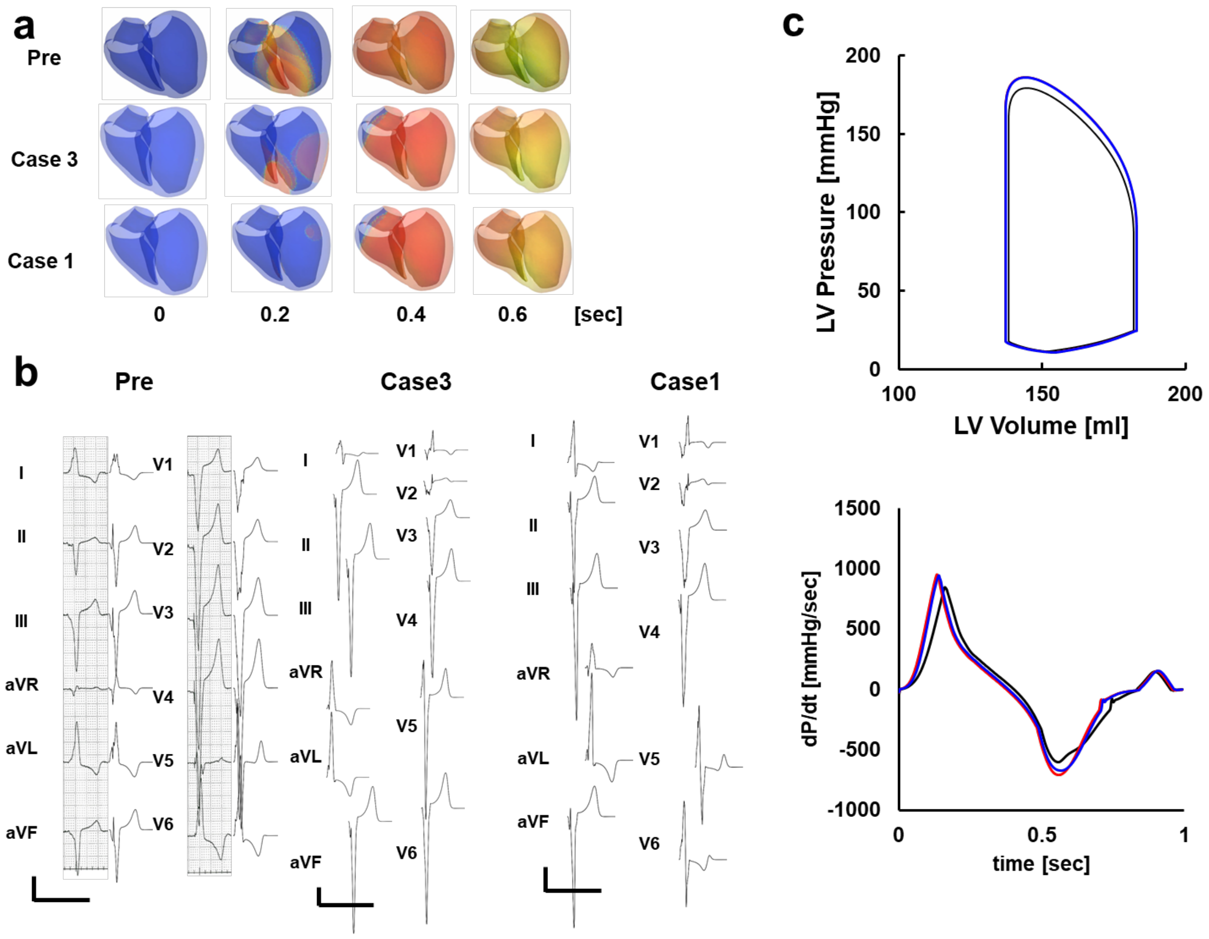

SFig.3 Patient-specific model and CRT effect in Patient #2

a: Time-lapse images showing the propagation of activation (color) and contraction before the treatment (Pre), with the lead position producing the best outcome (Case 3) and the worst outcome (Case 1). Numbers indicate the time after the onset of activation. b: Actual (left column) and simulated (right column) ECG under the three conditions. c: Pressure-volume loop of the left ventricle (upper panel) and dP/dt (lower panel) under the three conditions (Pre: black line, Case 3: red line, Case 1: blue line).

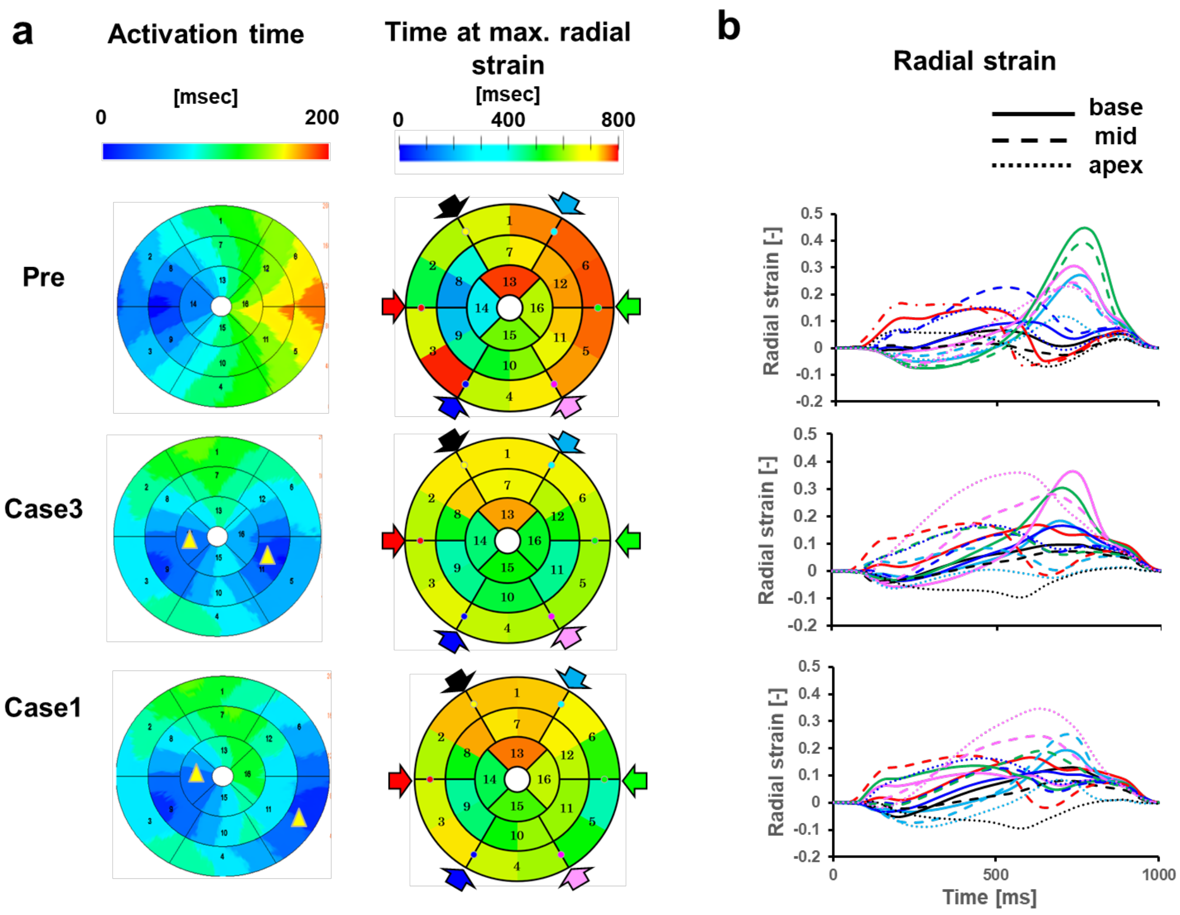

SFig.4 Evaluation of dyssynchrony

a: Activation time and time at maximum radial strain are shown by color in the bull's eye view of the left ventricle and compared among pre-CRT, case 3 and case 1. Yellow triangles indicate the approximate position of pacing sites. b: Temporal changes in radial strain, which was measured in 16 segments, compared among pre-CRT, case 3, and case 1. Data were sampled at the base (solid lines), mid-ventricular (broken lines) and apical (dotted lines) levels in six circumferential locations, which are indicated by color arrows in the bull's eye view. However, red and green were omitted at the apical level.

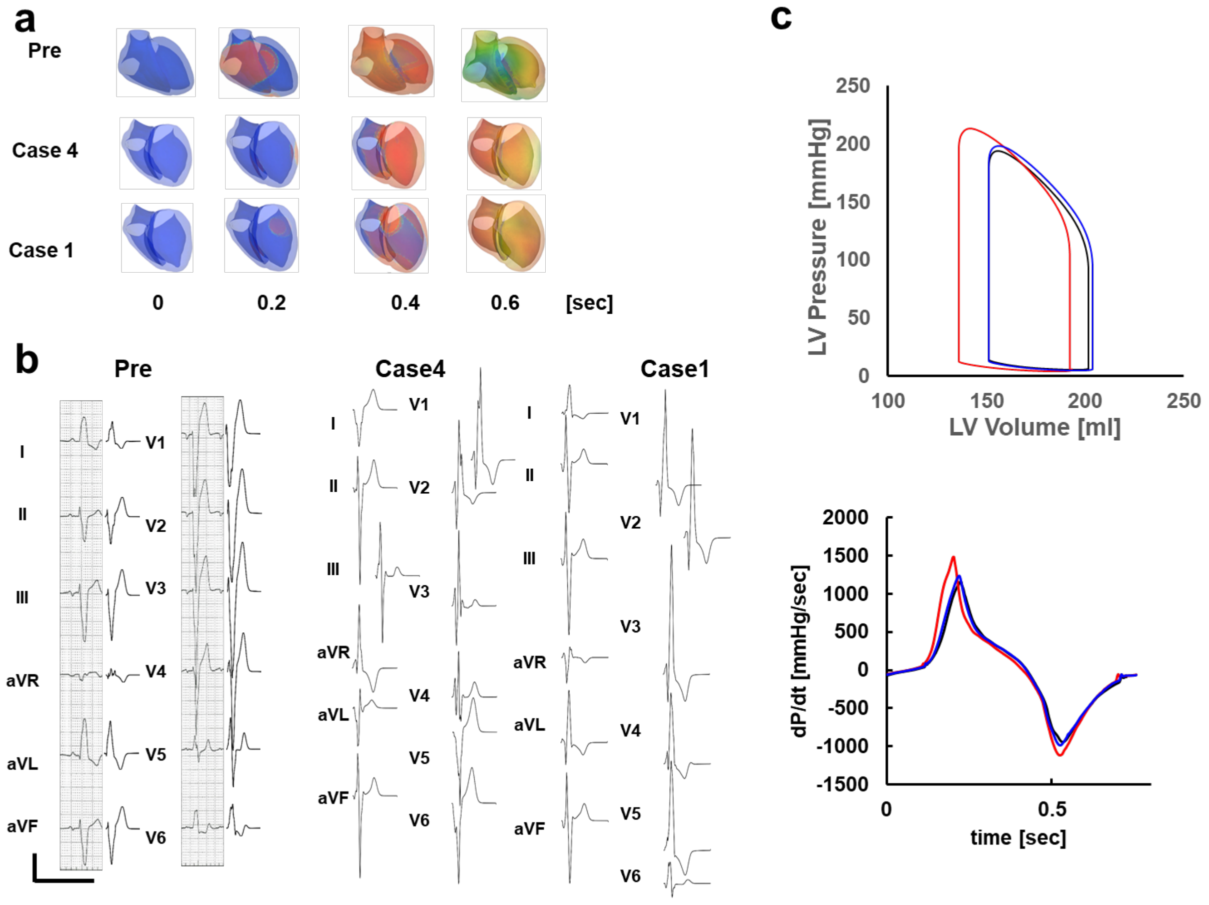

SFig.5 Patient-specific model and CRT effect in Patient #3

a: Time-lapse images showing the propagation of activation (color) and contraction before the treatment (Pre), with the lead position producing the best outcome (Case 4) and the worst outcome (Case 1). Numbers indicate the time after the onset of activation. b: Actual (left column) and simulated (right column) ECG under the three conditions. c: Pressure-volume loop of the left ventricle (upper panel) and dP/dt (lower panel) under the three conditions (Pre: black line, Case 4: red line, Case 1: blue line).

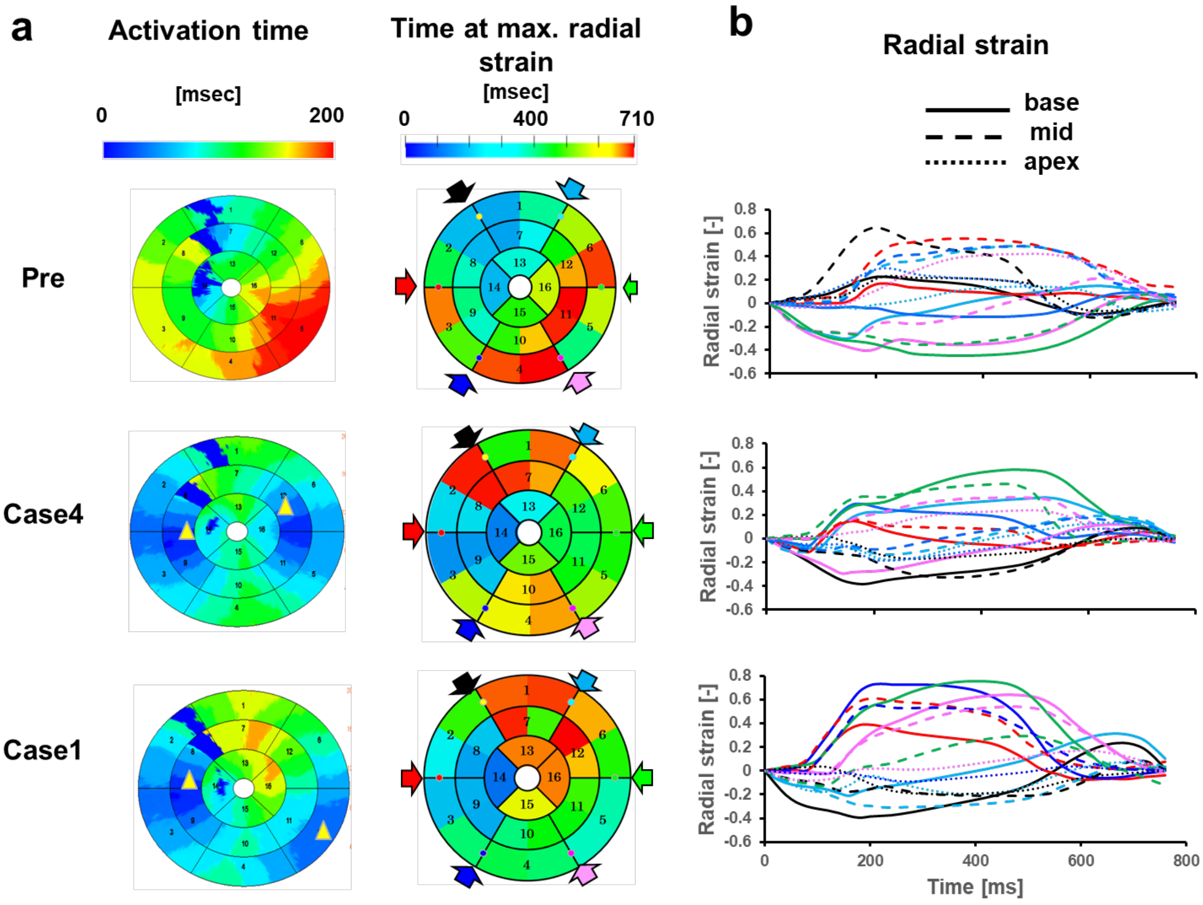

SFig.6 Evaluation of dyssynchrony

a: Activation time and time at maximum radial strain are shown by color in the bull's eye view of the left ventricle and compared among pre-CRT, case 4 and case 1. Yellow triangles indicate the approximate position of pacing sites. b: Temporal changes in radial strain, which was measured in 16 segments, compared among pre-CRT, case 4, and case 1. Data were sampled at the base (solid lines), mid-ventricular (broken lines) and apical (dotted lines) levels in six circumferential locations, which are indicated by color arrows in the bull's eye view. However, red and green were omitted at the apical level.

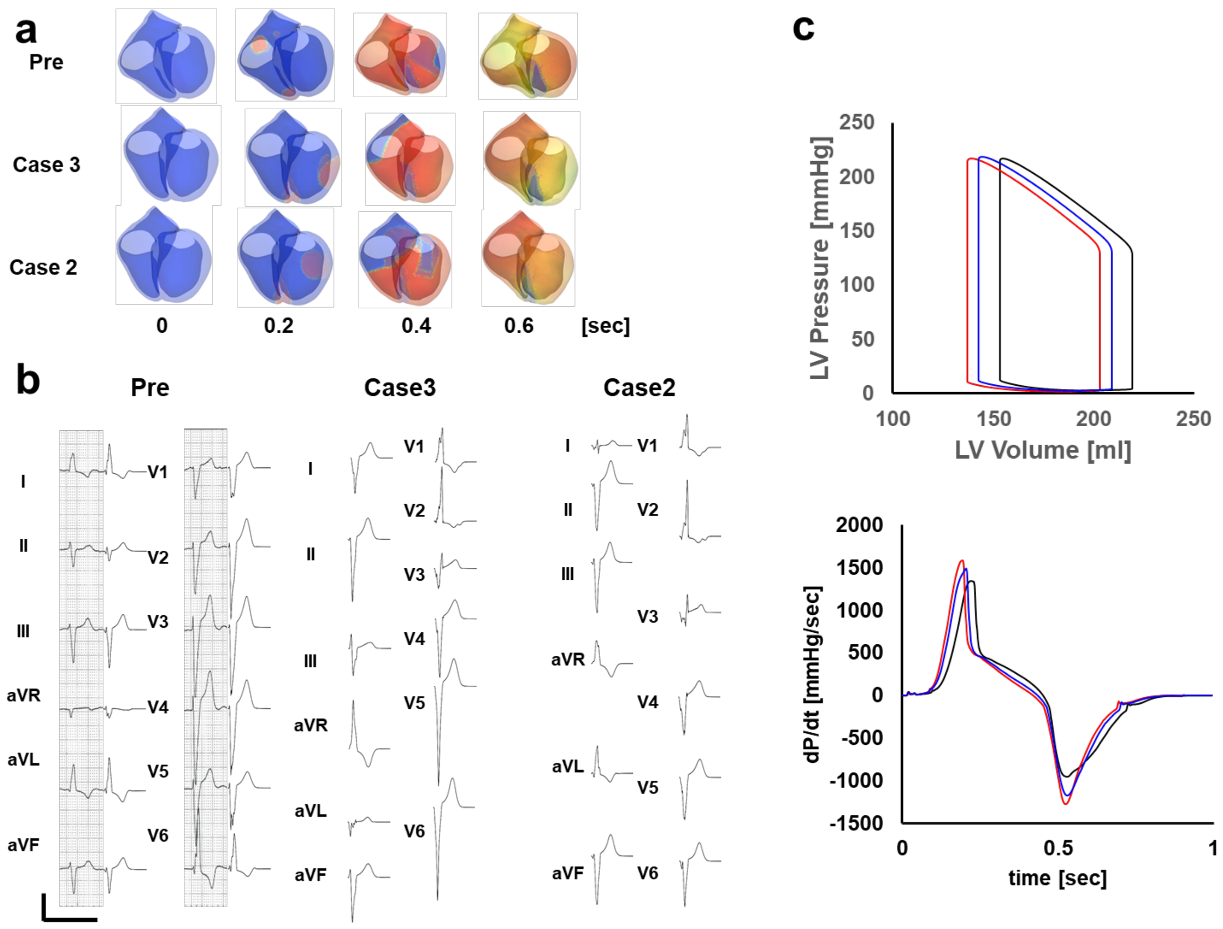

SFig.7 Patient-specific model and CRT effect in Patient #4

a: Time-lapse images showing the propagation of activation (color) and contraction before the treatment (Pre), with the lead position producing the best outcome (Case 3) and the worst outcome (Case 2). Numbers indicate the time after the onset of activation. b: Actual (left column) and simulated (right column) ECG under the three conditions. c: Pressure-volume loop of the left ventricle (upper panel) and dP/dt (lower panel) under the three conditions (Pre: black line, Case 3: red line, Case 2: blue line).

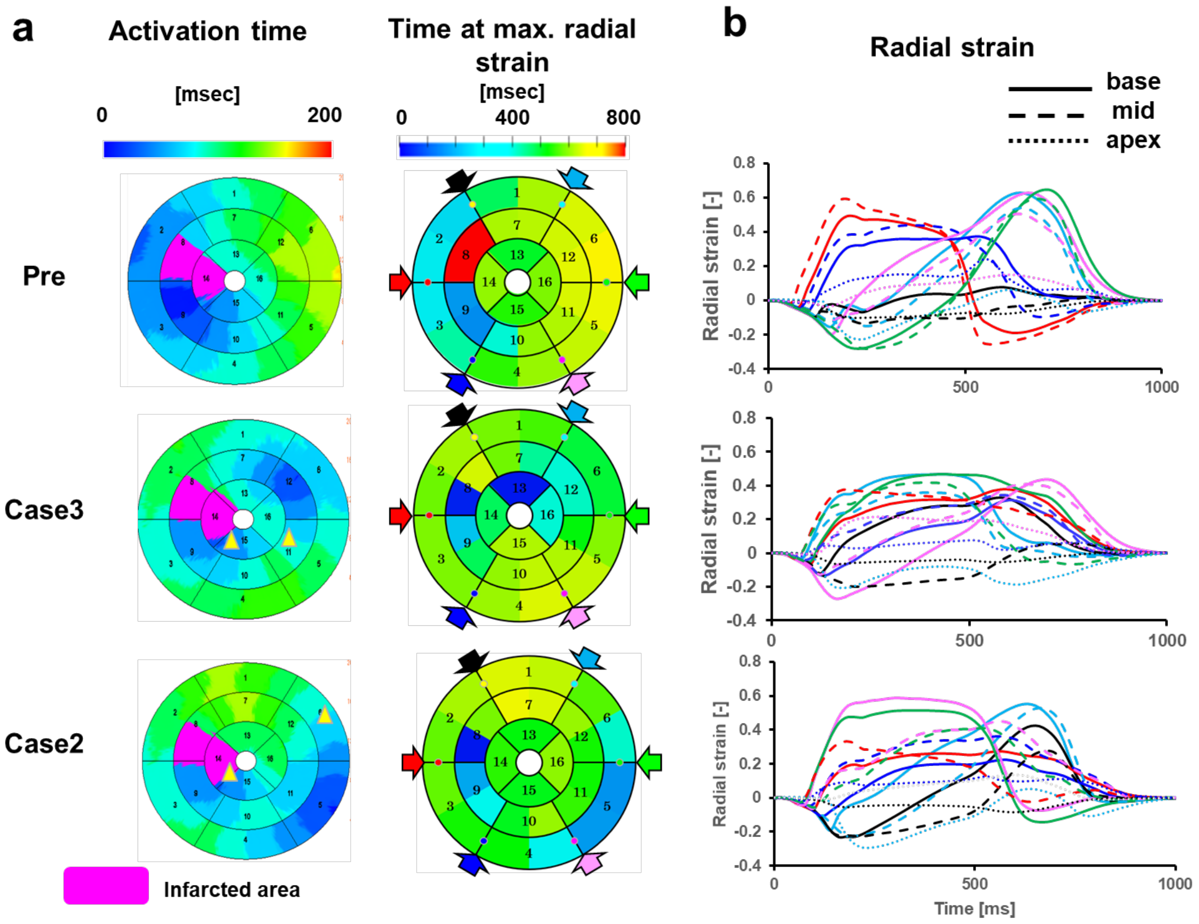

SFig.8 Evaluation of dyssynchrony

a: Activation time and time at maximum radial strain are shown by color in the bull's eye view of the left ventricle and compared among pre-CRT, case 3 and case 2. Yellow triangles indicate the approximate position of pacing sites. b: Temporal changes in radial strain, which was measured in 16 segments, compared among pre-CRT, case 3, and case 2. Data were sampled at the base (solid lines), mid-ventricular (broken lines) and apical (dotted lines) levels in six circumferential locations, which are indicated by color arrows in the bull's eye view. However, red and green were omitted at the apical level.

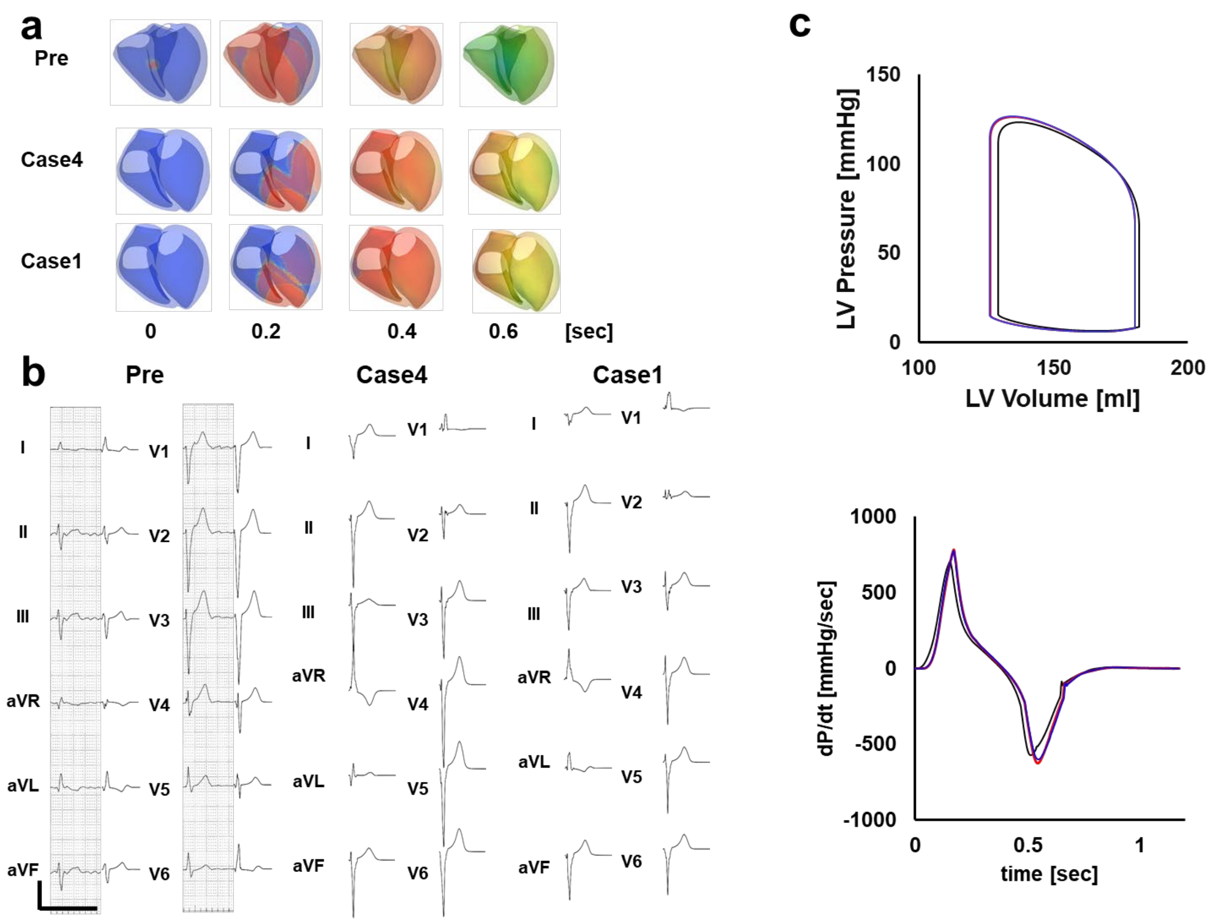

SFig.9 Patient-specific model and CRT effect in Patient #7

a: Time-lapse images showing the propagation of activation (color) and contraction before the treatment (Pre), with the lead position producing the best outcome (Case 4) and the worst outcome (Case 1). Numbers indicate the time after the onset of activation. b: Actual (left column) and simulated (right column) ECG under the three conditions. c: Pressure-volume loop of the left ventricle (upper panel) and dP/dt (lower panel) under the three conditions (Pre: black line, Case 4: red line, Case 1: blue line).

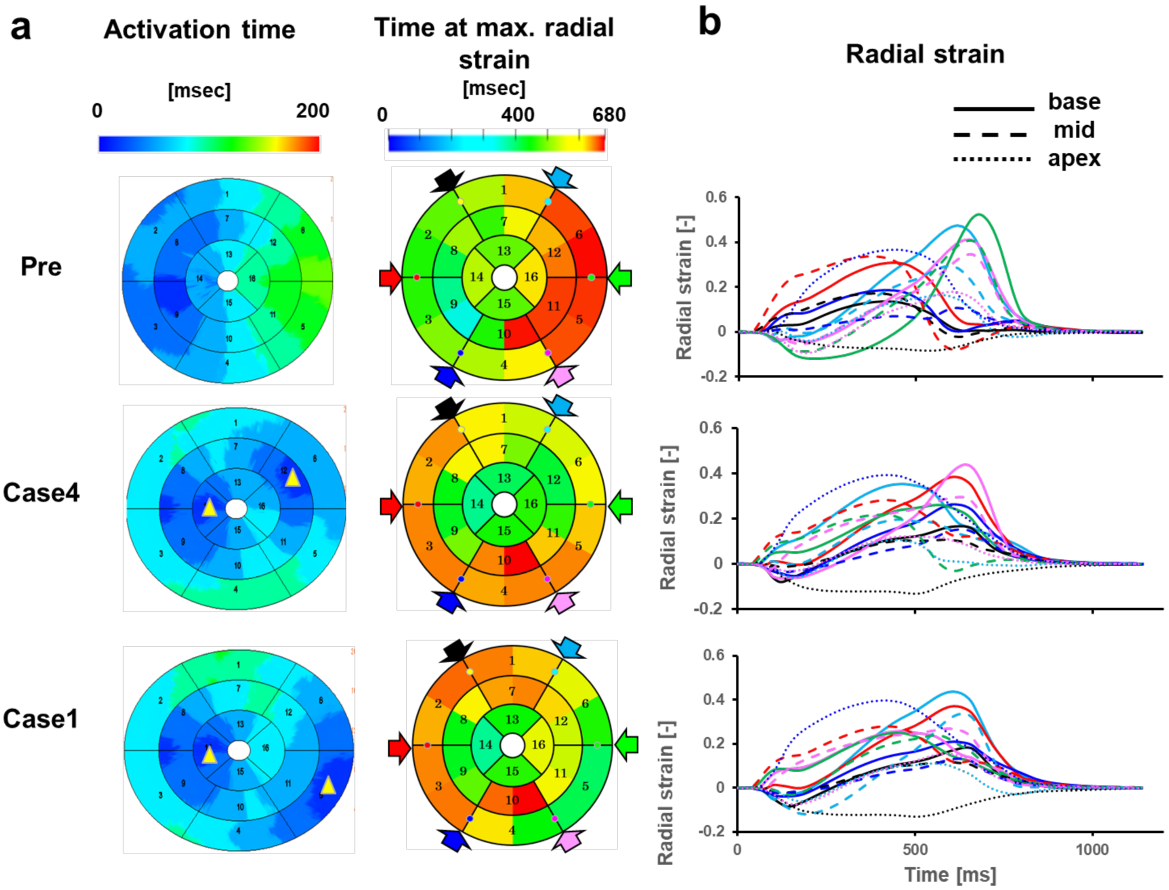

SFig.10 Evaluation of dyssynchrony

a: Activation time and time at maximum radial strain are shown by color in the bull's eye view of the left ventricle and compared among pre-CRT, case 4 and case 1. Yellow triangles indicate the approximate position of pacing sites. b: Temporal changes in radial strain, which was measured in 16 segments, compared among pre-CRT, case 4, and case 1. Data were sampled at the base (solid lines), mid-ventricular (broken lines) and apical (dotted lines) levels in six circumferential locations, which are indicated by color arrows in the bull's eye view. However, red and green were omitted at the apical level.

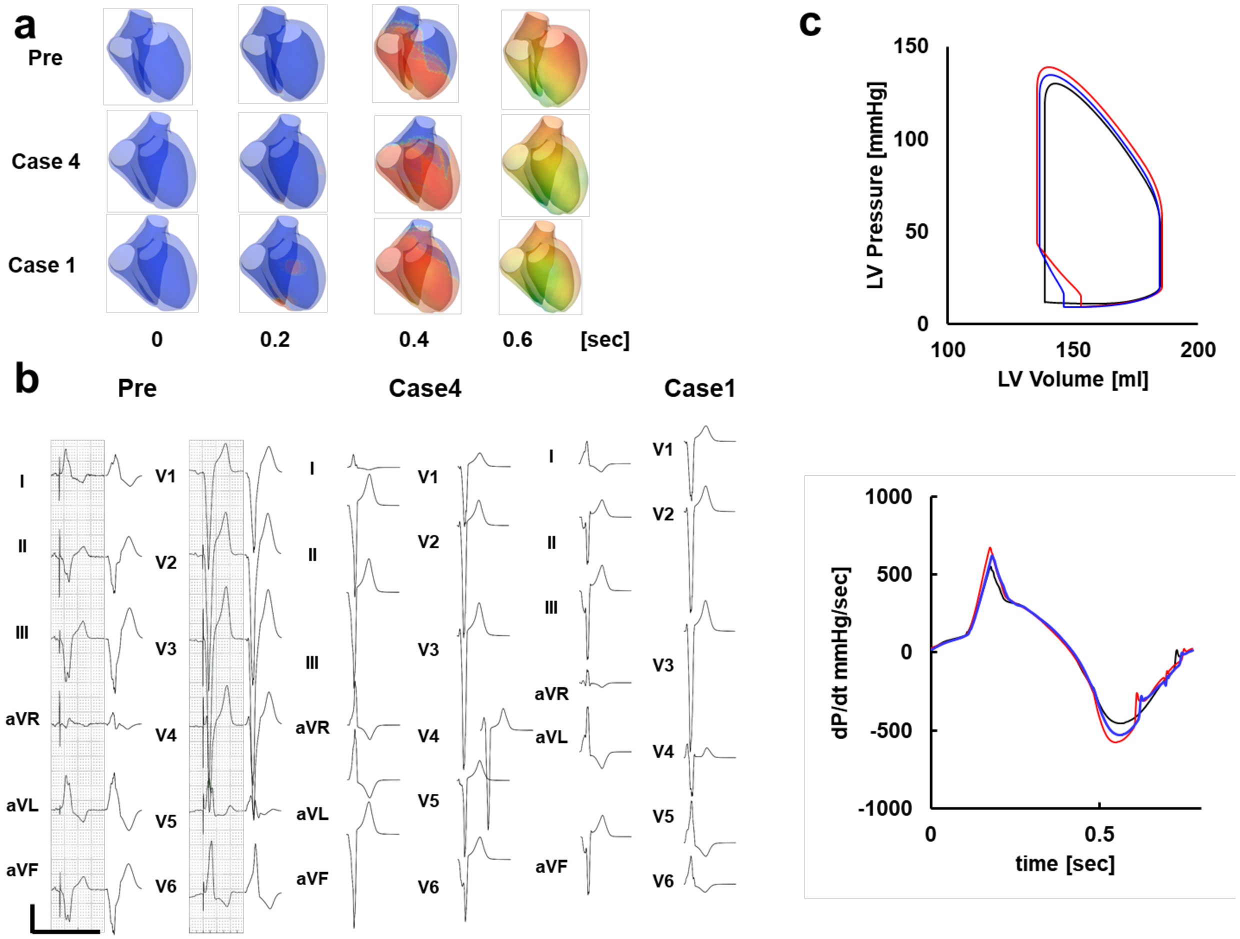

SFig. 11 Patient-specific model and CRT effect in Patient #8

a: Time-lapse images showing the propagation of activation (color) and contraction before the treatment (Pre), with the lead position producing the best outcome (Case 4) and the worst outcome (Case 1). Numbers indicate the time after the onset of activation. b: Actual (left column) and simulated (right column) ECG under the three conditions. c: Pressure-volume loop of the left ventricle (upper panel) and dP/dt (lower panel) under the three conditions (Pre: black line, Case 4: red line, Case 1: blue line).

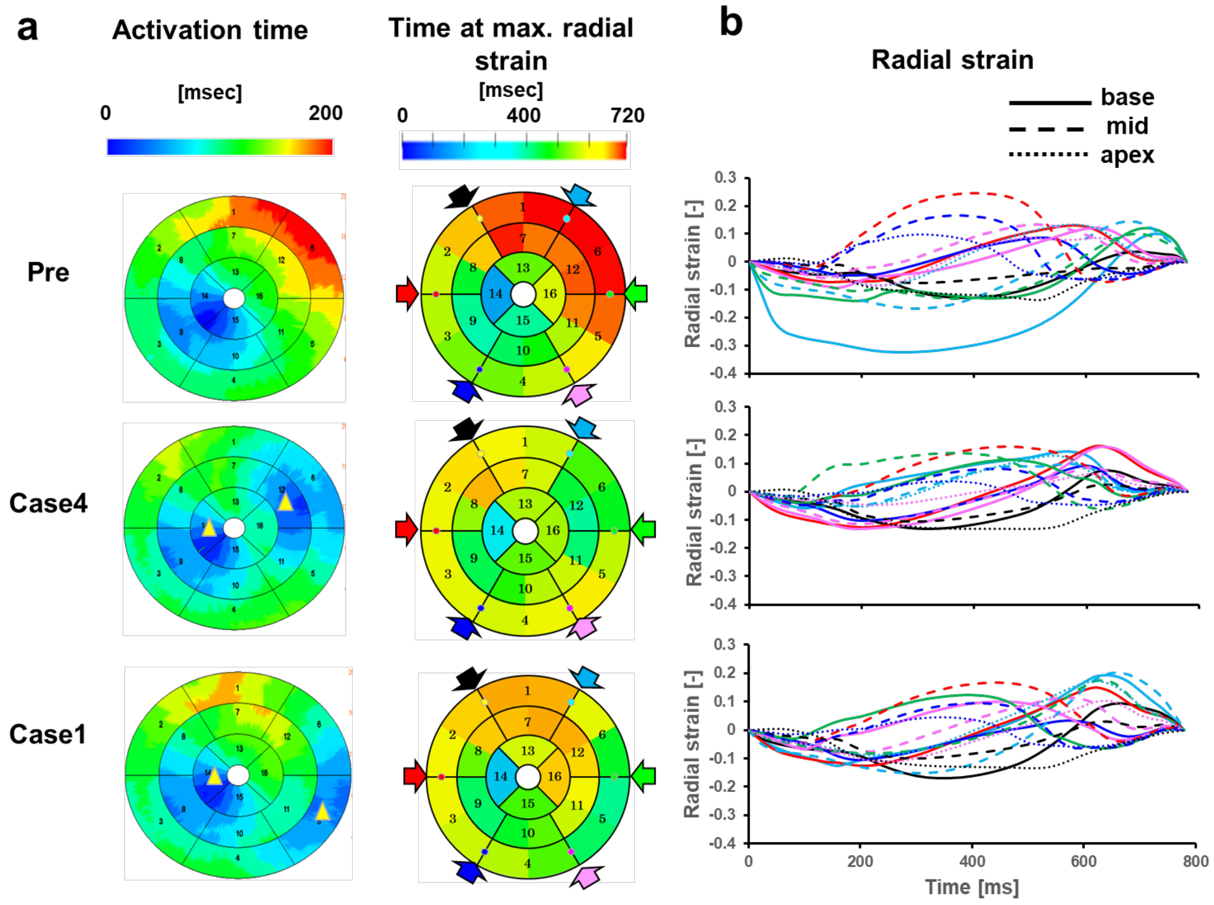

SFig. 12 Evaluation of dyssynchrony

aA: Activation time and time at maximum radial strain are shown by color in the bull's eye view of the left ventricle and compared among pre-CRT, case 4 and case 1. Yellow triangles indicate the approximate position of pacing sites. b: Temporal changes in radial strain, which was measured in 16 segments, compared among pre-CRT, case 4, and case 1. Data were sampled at the base (solid lines), mid-ventricular (broken lines) and apical (dotted lines) levels in six circumferential locations, which are indicated by color arrows in the bull's eye view. However, red and green were omitted at the apical level.
